# Genome-wide comparative analyses of GATA transcription factors among seven *Populus* genomes

**DOI:** 10.1038/s41598-021-95940-5

**Published:** 2021-08-16

**Authors:** Mangi Kim, Hong Xi, Suhyeon Park, Yunho Yun, Jongsun Park

**Affiliations:** 1 InfoBoss Inc., 301 room, Haeun Bldg., 670, Seolleung-ro, Gangnam-gu, Seoul, 07766 Korea; 2 InfoBoss Research Center, 301 room, Haeun Bldg., 670, Seolleung-ro, Gangnam-gu, Seoul, 07766 Korea

**Keywords:** Protein analysis, Gene regulatory networks, Sequence annotation

## Abstract

GATA transcription factors (TFs) are widespread eukaryotic regulators whose DNA-binding domain is a class IV zinc finger motif (CX_2_CX_17–20_CX_2_C) followed by a basic region. We identified 262 GATA genes (389 GATA TFs) from seven *Populus* genomes using the pipeline of GATA-TFDB. Alternative splicing forms of *Populus* GATA genes exhibit dynamics of GATA gene structures including partial or full loss of GATA domain and additional domains. Subfamily III of *Populus* GATA genes display lack CCT and/or TIFY domains. 21 *Populus* GATA gene clusters (PCs) were defined in the phylogenetic tree of GATA domains, suggesting the possibility of subfunctionalization and neofunctionalization. Expression analysis of *Populus* GATA genes identified the five PCs displaying tissue-specific expression, providing the clues of their biological functions. Amino acid patterns of *Populus* GATA motifs display well conserved manner of *Populus* GATA genes. The five *Populus* GATA genes were predicted as membrane-bound GATA TFs. Biased chromosomal distributions of GATA genes of three *Populus* species. Our comparative analysis approaches of the *Populus* GATA genes will be a cornerstone to understand various plant TF characteristics including evolutionary insights.

## Introduction

A transcription factor (TF) is a protein that controls the rate of transcription by binding to specific DNA sequences, including promoter regions. TF can also combine and interact with cis-acting elements in the promoter region as well as interact with other proteins to regulate the start site of transcription^1^. In plant, TF plays important roles such as controlling flower developments^2, 3^, circadian clock^4^, carbon and nitrogen regulatory networks^5^, protein–protein interaction^6^, cell differentiation^7^, pathogen and hormone responses^8^, and disease resistance^9^.

Due to a large number of plant genomes available (2,220 genomes from 725 species; Plant Genome Database Release 2.75; http://www.plantgenome.info/; Park et al., in preparation), many genome-wide analyses of plant TFs have been conducted^10–15^. One of the genome-wide TF databases is the plantTFDB which identifies 58 TF families from 165 plant species^11^. Some of these TF families are plant-specific, including AP2/ERF^16^, NAC^17^, WRKY^18^, and GRAS^19, 20^, and some are general in eukaryotic such as bHLH (basic helix-loop-helix)^21, 22^, bZIP (basic leucine-zipper)^23^, and GATA^24–27^. With the published plant genomes, various genome-wide analyses of TF families have been conducted; AP2/ERF, NAC^28–31^, bHLH^32–34^, bZIP^23, 35–37^, GRAS^20, 38, 39^, and GATA^25, 40, 41^ TF families displaying their features in various aspects including evolutionary aspect. Genome-wide analyses of TF families in *Arabidopsis thaliana* have also been studied, presenting 122 AP2/ERF genes^42^, 105 NAC genes^28^, 162 bHLH genes^34^, 75 bZIP genes^23^, 32 GRAS genes^20^, 29 GATA genes^25^ as well as 566 GATA genes from 19 *A. thaliana* genomes^43^.

GATA TFs contain more than one highly conserved type IV zinc finger motifs (CX_2_X_17–20_CX_2_C) followed by a basic region that can bind to a consensus DNA sequence, WGATAR (W means T or A; R indicates G or A)^25, 44, 45^. Most plant GATA TFs contain a single GATA domain of which pattern is CX_2_CX_18_CX_2_C (type IV_b_) or CX_2_CX_20_CX_2_C (type IV_c_)^27^. Except for these known types, additional patterns were also identified: e.g., CX_4_CX_18_CX_2_X, which have four amino acids in the first Cysteine-Cysteine, named as type IV_4_^43^.

Plant GATA TFs have various roles like the chloroplast development^46^, photosynthesis and growth^47^, epithelial innate immune responses^48^, seed germination^49^, hypocotyl and petiole elongation^50^, and cryptochrome1-dependent response^51^. Genome-wide analyses and/or expression analyses of GATA TFs have been reported in 21 plant species^25, 40, 41, 43, 52–66^ (Table S1), including *P. trichocarpa* of which genome-wide identification of GATA genes was conducted based on the old gene model (Version 3.0; Table S1).

*Populus* genus is a model system for investigating the wood development, crown formation, and disease resistance in perennial plants^67^, which has several advantages including rapid growth, ease of cloning, and small genome^68^. Owing to it, its genome was sequenced as a first wood plant genome^69^, and then additional genome sequences of *Populus* species have been sequenced and analyzed^55, 70–75^ (Table 1), which is an excellent resource to identify genus-wide analyses of *Populus* GATA TFs. These species were classified into independent three clades based on phylogenetic studies using single-copy^76^ nuclear genes and whole chloroplast genome sequences^77, 78^: (i) *P. tremuloides*, *P. tremula*, and *P. tremula* x *alba*, (ii) *P. pruinosa* and *P. euphratica*, and (iii) *P. trichocarpa* and *P. deltoides*. In spite of abundant resources of Salicaceae genomes including *Salix purpurea*^79^, there is only one study for characterizing the biological function of *Populus* GATA gene (*PdGNC*), which regulates chloroplast ultrastructure, photosynthesis, and vegetative growth in *Arabidopsis*^80^, suggesting genome-wide identification of *Populus* GATA genes are strongly required.

**Table 1:** Characteristics of identified GATA TFs from seven *Populus* genomes.

*Populus* species name	Version	# of GATA genes (A)	# of GATA TFs	# of GATA genes having alternative splicing forms (B)	# of GATA TFs having alternative splicing forms	# of genes	# of proteins	Ratio (B/A) (%)
*Populus trichocarpa*	3.1	39	67	13	41	42,950	63,498	33.33
*Populus pruinosa*	1	37	37	0	0	35,131	35,131	0.00
*Populus euphratica*	1	40	55	9	24	30,688	49,676	22.50
*Populus deltoides*	2.1	38	55	7	24	44,853	57,249	18.42
*Populus tremuloides*	1.1	37	44	7	14	36,830	48,320	18.92
*Populus tremula*	1.1	33	60	16	43	35,309	83,720	48.48
*Populus tremula* x *alba*	1.1	38	71	16	49	41,335	73,013	42.11
Total		262	389	68	195	267,096	410,607	30.22*

*It shows that total ratio (B/A) of *Populus* genomes except *P. pruinosa*.

Here, we conducted genome-wide identification, phylogenetic analyses, expression level analysis, identification of amino acid patterns and transmembrane helix of GATA TFs in seven *Populus* genomes with the GATA-TFDB (http://gata.genefamily.info/; Park et al., in preparation). Our comparative and comprehensive analyses conducted with the integrated bioinformatic pipeline provided by the GATA-TFDB will be a cornerstone to understand various plant TF characteristics including evolutionary insights.

## Results and discussions

### Identification of GATA TFs from seven *Populus* genomes

**Figure 1:**
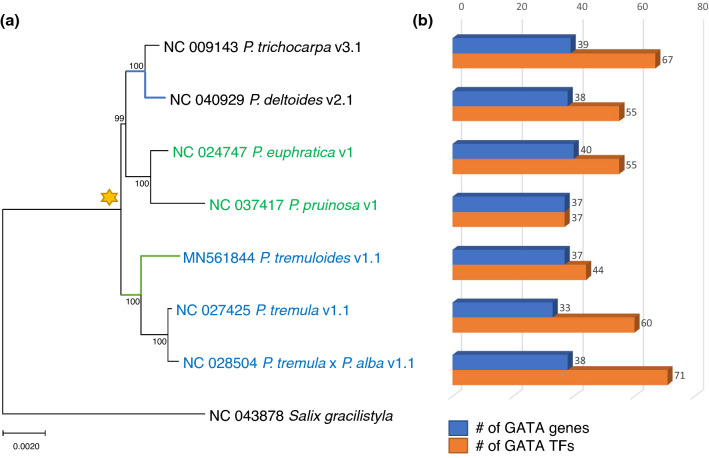
Phylogenetic tree of seven *Populus* species in complete chloroplast genomes. (**a**) shows a phylogenetic tree of seven *Populus* species in complete chloroplast genomes. Bootstrap analyses with 1000 pseudo-replicates were conducted with the same options. *S. gracilistyla* is aligned to the seven *Populus* genomes as an outgroup. Light blue and green lines indicate the gene loss of GATA TFs in *P. deltoides* and *P. tremuloides* lineage, respectively. Yellow star means gene duplication events of the 10 paralogous pairs. Black, green, blue letters of 7 *Populus* genomes mean independent three clades based on phylogenetic studies. (**b**) presents the number of GATA genes and GATA TFs for each genome.

We identified 262 GATA genes (389 GATA TFs) from seven *Populus* genomes available in public using the pipeline of the GATA-TFDB (http://gata.genefamily.info/; Table S2). The number of GATA genes for each *Populus* genome ranges from 33 to 40 (Table 1), which is larger than those of *A. thaliana* (29 to 30 GATA genes)^25, 43^, *V. vinifera* (19 GATA genes), and *O. sativa* (28 GATA genes)^81^; while is smaller than that of *G. max* (64 GATA genes)^40^. The phylogenetic relationship of the seven *Populus* species inferred from the complete chloroplast genomes (Fig. 1a), congruent to the previous studies^76, 78^, shows no correlation with the number of GATA genes. It can be explained that the number of GATA genes is rather affected by the accuracy of the gene model (e.g., the largest number of GATA TFs is not from *P. trichocarpa*, which is the model *Populus* species; Fig. 1b). The proportion of *Populus* GATA genes against whole genes ranges from 0.08% (*P. deltoides*) to 0.13% (*P. euphratica*; Table 1), which is similar to that of *A. thaliana* (0.11%) and is slightly higher than those of *V. vinifera* (0.07%), *G. max* (0.06%), and *O. sativa* (0.05%).

Among *Populus* genomes, *P. euphratica* has the largest number of GATA genes (40); while *P. tremula* contains the smallest (33; Fig. 1b): difference of the number of GATA genes between the largest and the smallest is seven. Similarity, three *Arabidopsis* genomes, *Arabidopsis halleri*, *Arabidopsis lyrata*, and *A. thaliana*, contain 22, 28, and 30 GATA genes, respectively (Fig. S1) and four *Oryza* genomes, *O. sativa*, *Oryza glaberrima*, *Oryza brachyantha*, and *Oryza rufipogon*, showed 28, 25, 24, and 28 GATA genes, respectively^25^ (Fig. S1), displaying similar interspecies differences. While, *Gossypium raimondii*, *Gossypium arboretum*, and *Gossypium hirsutum* presented 46, 46, and 87, respectively because *G. hirsutum* is a tetraploid species^52^. These interspecific differences of GATA genes indicate that many evolutionary events including the gain and loss of GATA genes were occurred in three genera.

Except *P. pruinosa* genome not containing alternative splicing forms, numbers of GATA TFs are larger than those of GATA genes (Table 1). Numbers of GATA genes which have alternative splicing forms range from 7 to 16 (Table 1 and Table S3), accounting for 30.22% of *Populus* GATA genes, which is similar that of *A. thaliana* (9 out of 30 GATA genes; 30.00%). PdGATA6 from *P. deltoides* contains nine alternative splicing forms, which is the largest number. In addition, *P. tremula* (PtaGATA26) and *P. trichocarpa* (PtrGATA12) show seven, *P. euphratica* (PeGATA35) displays six, *P. tremula* x *alba* (PtaaGATA36) presents five, and *P. tremuloides* (PtsGATA3, 17, 22, 27, 30, 35, and 36) has two. Average numbers of alternative splicing forms of GATA genes range from 2.00 (*P. tremuloides*) to 3.43 (*P. deltoides*). These differences can be partially explained by that alternative splicing forms are controlled via multilayered regulatory network^82^, however, further studies are required.

Differences in the number of alternative splicing forms of GATA genes in *Populus* genus can be caused by (i) different gene prediction programs^83–85^ and (ii) amount of evidence transcript sequences, covering fully characterized genes, expressed sequences tags (ESTs), and/or RNA-Seq data. In the gene prediction process, evidence sequences are essential to achieve accurate prediction of genes as well as alternative splicing forms. More available EST or RNA-Seq sequences will bring plentiful alternative splicing forms of GATA genes: e.g., the human genome contains around averagely of 10 alternative splicing forms per one gene, predicted from a large amount of transcript sequences^86^. Available RNA-Seq data of *Populus* genus (As of 2018 Jun) deposited in NCBI Short Read Archive show that *P. trichocarpa* and *P. tremula* presenting a large proportion of alternative splicing forms of GATA genes contain a large amount of RNA-Seq data (Table S4).

### In-depth investigations of alternative splicing forms of *Populus* GATA genes

**Figure 2:**
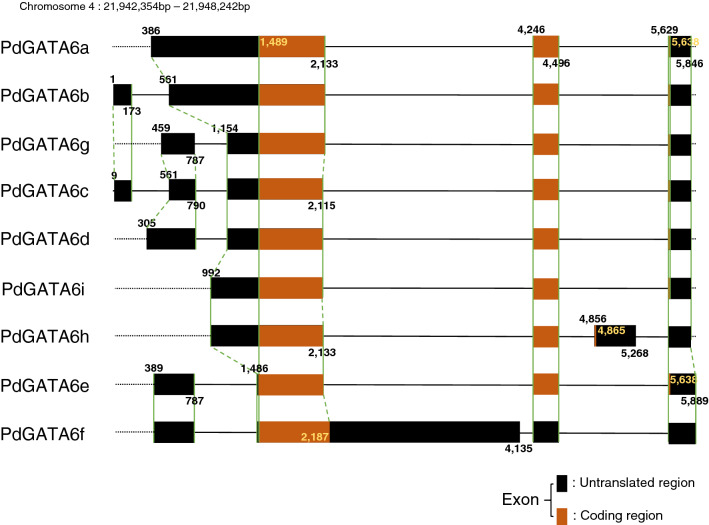
Diagram of alternative splicing forms of PdGATA6. It shows the gene structure of the PdGATA6 gene (*P. deltoides*). Orange thick boxes indicate translated regions and black thick boxes display untranslated regions. Black thin lines mean intron and black dotted lines are intergenic regions. Green dotted and solid lines indicate the conserved and different structure of GATA genes including exon, intron, and, untranslated regions, respectively. The number around the boxes display relative positions of translated, untranslated, and exons based on the start position of PdGATA6b. Names of alternative splicing forms of the PdGATA6 gene are displayed in the left part of each gene diagram.

We identified the phenomena that some alternative splicing forms originated from one *Populus* GATA gene encode the same amino acids. Each of the nine alternative splicing forms of PdGATA6, a typical example of this phenomenon, is composed of 232 aa, 295 aa, and 301 aa in protein length and exon is composed of between 3 and 5. Among the nine alternative splicing forms of PdGATA6, all except PdGATA6f and 6h present the same start and end positions of ORFs. The first ORF exons of the eight alternative splicing forms except PdGATA6f contain start methionine without stop codon are classified into two types: one is 627 bp and the other is 645 bp. It results in two types of amino acid sequences from the eight alternative splicing forms, indicating that most of alternative splicing events are occurred in 5’ and 3’ UTR regions (Fig. 2). In addition, GATA domain sequences of eight alternative splicing forms of PdGATA6 are identical, suggesting that the GATA domain is important to bind DNA.

Interestingly, some of alternative splicing forms of *Populus* GATA genes display the same amino acids: one GATA gene from *P. tremuloides*, four from *P. euphratica* and *P. deltoides*, six from *P. trichocarpa* and *P. tremula* x *alba*, and eight from *P. tremula*. One of known roles of untranslated regions of messenger RNA is changing the amount of translated proteins^87^. The number of TFs will increase or decrease the transcription amount of target genes, so that these alternative splicing forms may be important to the regulatory network of GATA TFs.

We also identified that some alternative splicing forms of the twelve GATA genes of three *Populus* species (*P. tremula* x *alba*, *P. tremula*, and *P. tremuloides*; Table S5) missed GATA domain which was not included in the *Populus* GATA TFs list. Interestingly, GATA TFs without GATA domain can negatively regulate the target genes by competing with normal GATA TFs^88^, indicating that these twelve GATA genes containing alternative splicing forms without domain can also play a role of negative regulators. In addition, one *Populus* GATA gene, PtaGATA28 (from *P. tremula*), has five out of six alternative splicing forms that missed GATA domain, suggesting that this gene might have a dominant role of negative regulation in contrast to the normal GATA genes even though additional research such as expression level of each alternative splicing forms in various conditions are required. PtaGATA33 from *P. tremula* has two out of three, and the rest ten GATA genes have one.

### Identification and investigation characteristics of *Populus* GATA subfamilies

**Figure 3:**
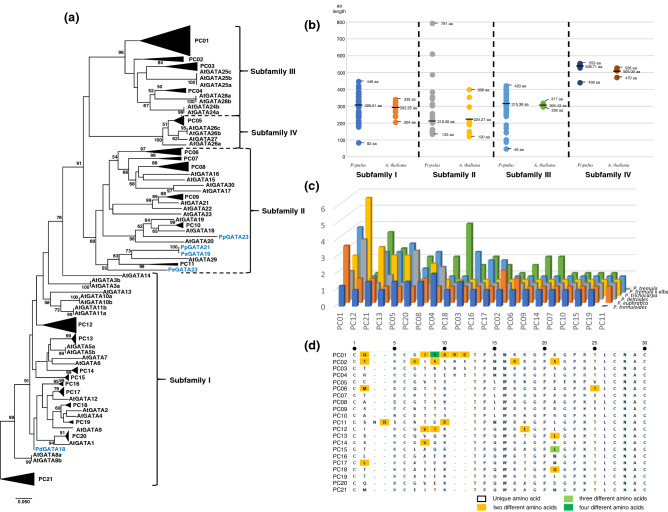
Sequence characteristics of *Populus* GATA TFs. (**a**) shows that the phylogenetic tree of GATA domain sequences constructed by the neighbor-joining method. Black triangles on the tree indicate a group of *Populus* GATA domains. Names of the *Populus* GATA genes not condensed were displayed with blue colors. Bootstrap values calculated from 10,000 replicates are shown on the node except that those are lower than 50. The scale bar corresponds to 0.050 estimated amino acid substitutions per site. Ranges of subfamilies were presented with lines on the right side. (**b**) displays distribution of amino acid length of *Populus* and *A. thaliana* GATA TFs. The length distribution of GATA genes in each subfamily was plotted separated with the dotted lines. The Y-axis represents an amino acid length of GATA TF. Bold lines mean average length of GATA genes. Thin lines present maximum or minimum the length of GATA genes. (**c**) provides the information of conserved and diversity splicing forms of GATA genes among six *Populus*. The X-axis displays a list of PCs. Y-axis indicates *Populus* species name. Z-axis shows the ratio of the number of GATA TF per GATA gene. (**d**) presents the pattern of amino acid sequence of GATA motif (CX_2-4_CX_18-20_CX_2_C) along with *Populus* gene clusters (PCs). Yellow, light green, and dark green shaded alphabets mean two, three, and four different amino acids in that position, respectively.

We constructed a neighbor-joining phylogenetic tree based on amino acid sequences of GATA domains of 389 *Populus* and 41 *Arabidopsis* GATA TFs together to identify subfamilies (Fig. 3a), resulting those four subfamilies (I to IV; see Material and Methods; Table S2) were successfully identified. Subfamily I has the largest number of *Populus* GATA genes; while subfamily IV contains the smallest number (Table S6) as same as *A. thaliana*^25^, *V. vinifera*^89^, and *G. max*^40^ except *O. sativa*^81^, monocot species. Subfamily III presents the largest average number of alternative splicing forms (1.81) and subfamily II displays the lowest (1.15; Table S6). GATA domains belonging to subfamilies I, II, and III are located adjacent to the C-terminal; however, those in subfamily IV are at the N-terminal as same as *A. thaliana*^25^, *V. vinifera*^89^, *G. max*^40^, and *O. sativa*^81^.

Amino acid lengths of *Populus* GATA TFs in each subfamily present a wider range than those of *A. thaliana* (Fig. 3b). However, three *Populus* GATA TFs display extremely short lengths: PdGATA18 belonging to subfamily I is 82 aa, PtsGATA29 and PdGATA36 from subfamily III are 46 and 86 aa, respectively (Table S2). Interestingly, some of GATA TFs of the other plant species including *A. thaliana* also display the short GATA TFs: 120 aa (AtGATA23) in *A. thaliana*^25^, 109 aa (VvGATA13) in *V. vinifera*^89^, 80 aa (GmGATA10) in *G. max*^40^, and 101 aa (OsGATA8b) in *O. sativa*^81^. It indicates that the three shortest *Populus* GATA TFs may be functional GATA TFs, suspecting that the gene prediction program can miss some of exons nearby the exon containing the GATA domain.

Two *Populus* GATA TFs in subfamily II have unique domains in comparison to those of *A. thaliana*; PpGATA21 contains NIR domain (IPR005343) found in the Noc2 gene family in *Arabidopsis*. This domain seems to be involved in protein–protein interaction, indicating that PpGATA21 may have partner protein for forming protein complex to regulate target genes. In addition, PpGATA23 covers two HMA domains (IPR006121), which can bind heavy metal ions^90^. These two *Populus* GATA genes will have unknown additional functions like WC1, which is involved in circadian clock mechanism of *Neurospora crassa* with light-sensing domain^91^.

*A. thaliana* GATA TFs in subfamily III contain two known additional domains: CCT domain (IPR010402) found in circadian clock and a flowering control gene (CONSTANS^92^) and TIFY domain (IPR010399) to mediate homo- and heteromeric interactions between TIFY proteins and other specific TFs^93, 94^. In contrast to *A. thaliana*^25^ and *G. max*^40^, fourteen GATA TFs in six *Populus* species except *P. euphratica* lack CCT and/or TIFY domains. Some of GATA TFs of *O. sativa* also presented the same phenomenon^81^. In detail, nine of the 14 GATA TFs are the unique transcript, indicating that they completely lost CCT and/or TIFY domains during evolution, similar to those of *V. vinifera*^89^. The remaining five GATA TFs have other alternative splicing forms, presenting the selective loss of these domains.

### Comparisons of the principal component analysis of seven *Populus* species

In a previous study, six *Populus* species except *P. tremula* x *alba* were used for conducting the phylogenetic analysis based on a total of 76 morphological properties for buds, leaves, inflorescences, flowers, and fruits^95^, which is congruent to the chloroplast phylogenetic tree (Fig. 1), except *P. trichocarpa* and *P. deltoides*. The incongruency of the two species is caused by limited species in Fig. 1. However, the principal component analysis of *Populus* GATA genes shows one cluster covering *P. euphratica*, *P. tremula* x *alba*, *P. tremuloides*, and *P. trichocarpa* (Fig. S2), which is incongruent to the aforementioned two phylogenetic trees. It reflects that GATA TFs may not evolve in the similar to species evolution due to their widely regulatory roles^96^.

### Identification of *Populus* GATA gene clusters (PCs) on phylogenetic tree of *Populus* GATA genes

To understand the phylogenetic relationship of *Populus* GATA genes clearly, we clustered them in the phylogenetic tree (Fig. 3a), resulting in 21 distinct *Populus* GATA gene clusters (PCs; Fig. 3a and Table S7). All PCs except PC11 containing five species except *P. tremuloides* and *P. tremula* covers all seven *Populus* species, displaying conserveness of *Populus* GATA genes. Eleven of the 21 PCs (52.38%) contain the same amount of GATA genes for each *Populus* species, while the rest 9 PCs (42.86%) show different numbers (Table S8). PC03, PC05, and PC14 lack of only one GATA gene from *P. pruinosa*, *P. tremula*, and *P. deltoides*, respectively; while PC12 and PC20 have additional GATA gene of *P. deltoides* and *P. euphratica*, respectively. The remaining 4 PCs display a complex pattern of the number of GATA genes for each *Populus* species (Table S8), indicating complex history of gain and loss of GATA genes in the *Populus* genus.

Subfamily I, containing the largest *Populus* GATA genes, displays the most complex structure with the largest number of PCs (Fig. 3a). Interestingly, AtGATA3, AtGATA10, AtGATA11, AtGATA13, AtGATA14, and PC12 do not show neighbor GATA genes like an independent clade (Fig. 3a). Subfamily II shows the largest ratio of the number of PCs to the number of GATA genes, implying faster evolution might be occurred. In addition, four *Populus* GATA genes (PpGATA21, PpGATA23, PeGATA19, and PeGATA23) are not clustered into PCs (Fig. 3a), presenting species-specific GATA genes. In subfamily III, PC01, containing four *Populus* GATA genes per species, might be experienced a gene duplication event in comparison to the other PCs. In addition, PC01 and PC02 seem to be independent of three *Arabidopsis* GATA genes (Fig. 3a), suggesting *Populus*-specific GATA genes, while PC03 and PC04 have their partner *Arabidopsis* GATA genes (Fig. 3a). Subfamily IV covers only one PC and two *Arabidopsis* GATA genes, the smallest subfamily (Fig. 3a).

Among six *Populus* species except *P. pruinosa*, PCs that have a relatively high average of the ratio of the number of GATA TF per GATA gene are PC01 (2.58), PC12 (2.36), and PC21 (2.17) (Fig. S3), suggesting that GATA TFs in these PCs may have diverse biological functions, similar to the case of OsGATA23^81^. In the species level, PC01 in *P. euphratica* (3.50) and *P. tremula* x *alba* (4.00), PC12 in *P. deltoides* (3.67) and *P. trichocarpa* (6.00), PC04 in *P. tremuloides* (2.00), and PC18 in *P. tremula* (4.00) display the high ratio, while five PCs (PC07, PC10, PC11, PC15, and PC19) have one GATA TF per GATA gene (Fig. 3c). We suspected that GATA TFs of each *Populus* species might have dynamic features of their functional diversification, including subfunctionalization and neofunctionalization^97–99^.

In total, 23 out of 556 (4.14%) amino acid positions in the CX_2-4_CX_18-20_CX_2_C region (inter-species variations) show more than one amino acid (Fig. 3d), which is larger than that of 19 *A. thaliana* genomes^43^ (intraspecific variations; 0.93%). Positions of variable amino acids in the CX_2-4_CX_18-20_CX_2_C region are scattered throughout this region along with PCs (Fig. 3d). Most variable positions are 9th in PC01 (four amino acids) and 21^st^ in PC15 (three amino acids; Fig. 3d); while the maximum number of different amino acids in *Arabidopsis* GATA genes is 2^43^.

### Genome-wide inference of GATA TF functions based on characterized *A. thaliana* GATA TFs

Till now, biological functions of one *Populus*^80^ and 15 *A. thaliana* GATA TFs have been characterized^43^. Nine of 15 characterized *Arabidopsis* GATA genes were also successfully mapped based on the PCs (Fig. 3a and Table 2) and similarity of amino acids, resulting in that seven PCs are related to the characterized *Arabidopsis* GATA genes. 129 *Populus* GATA TFs in the seven PCs are candidates for deducing their functional roles in *Populus*. In addition, *PdGNC* from *P. nigra* x (*P. deltoides* x *P. nigra*), a uniquely characterized GATA TF, known to regulate chloroplast ultrastructure, photosynthesis, and vegetative growth in *Arabidopsis*^80^ is successfully mapped to PtrGATA19 (PC09) with 98.02% amino acid similarity. It supports this inference method because both *GNC* in *Arabidopsis* and *PdGNC* are in the same PC with the similar functions even though *Arabidopsis* and *Populus* belong to Brassicaceae and Salicaceae and are an herb and a tree species, respectively. Moreover, OsGATA12 involved in the seedling stage based on expression profile, is similar to that of *BME3* in *A. thaliana*^81^. Based on this result, researchers can efficiently and systematically identify the biological functions of *Populus* GATA genes in the near future.

**Table 2:** Function inference of *Populus* GATA gene clusters (PCs) based on characterized *A. thaliana* GATA TFs.

PC name	GATA gene	Biological functions	Subfamily	References
PC03	AtGATA25	Hypocotyl and petiole elongation	III	^50^
PC04	AtGATA28 (ZML2)	Mediation of cryptochrome1-dependent response		^133^
PC08	AtGATA15 (GATA15)	Cytokinin-regulated development, including greening, hypocotyl elongation, phyllotaxy, floral organ initiation, accessory meristem formation, flowering time, and senescence	II	^135^
	AtGATA16 (GATA16)			
PC09	AtGATA21 (GNC)	a nitrate‐inducible member important for chlorophyll synthesis and glucose sensitivity		^136^
		Modulation of chlorophyll biosynthesis (greening) and glutamate synthase (GLU1/Fd-GOGAT) expression		^137, 138^
		Downstream effectors of floral homeotic gene action by controlling two MADS-box TFs		^139^
		Control of convergence of auxin and gibberellin signaling		^140, 141^
		Control of greening, cold tolerance, and flowering time		^142^
		Regulation of chloroplast development, growth, and division as well as photosynthetic activities		^143, 144^
		Cytokinin-regulated development, including greening, hypocotyl elongation, phyllotaxy, floral organ initiation, accessory meristem formation, flowering time, and senescence		^135^
		PIF- and light-regulated stomata formation in hypocotyls		^145^
PC10	AtGATA18 (HAN)	Regulation of shoot apical meristem and flower development		^145–149^
		Stable establishment of cotyledon identity during embryogenesis		^148^
		Position the proembryo boundary in the early *Arabidopsis* embryo		^149^
PC19	AtGATA2 (GATA-2)	Regulation of light-responsive genes	I	^45^
	AtGATA4 (GATA-4)			
PC20	AtGATA1 (GATA-1)			

### Expression level analysis of GATA genes in *P. deltoides* and *P. pruinosa*

**Figure 4:**
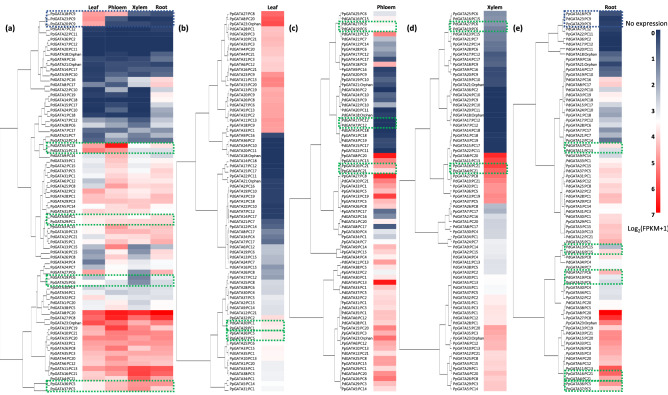
Heatmap of GATA genes in four tissues of *P. deltoides* and *P. pruinosa*. Dendrograms at the left side of heatmaps are the result of hierarchical clustering of each expression data using hclust function in R package stats version 4.0.3. Heatmaps present expression levels based on FPKM values calculated by cufflink with gradient colors from blue to red displayed in the legend on the right side. Dendrograms at the left side of heatmaps are the result of hierarchical clustering of each expression data. Labels consist of GATA gene and PC names separated with ‘:’. Green dotted boxes indicate the case that some of members in the same PC were clustered. Blue dotted boxes mean that all members in the PC were clustered together. (**a**) displays heatmap of GATA genes of two *Populus* species in the four tissues, (**b**)–(**e**) are for heatmaps in leaf, phloem, xylem, root tissues.

Based on available RNA-Seq raw reads obtained from leaf, phloem, xylem, and root tissues of *P. deltoides* and *P. pruinosa* (Table S9), expression levels of *Populus* GATA TFs were calculated (Fig. 4). In the four tissues, three GATA genes in the PC9 were well clustered displaying leaf and phloem specific expressions (Fig. 4a), which is congruent to the putative functions of GATA genes in PC9, such as regulation of chloroplast development, growth, and divisions (Table 2). In addition, four clusters covering GATA genes in PC13, PC1, PC6, and PC5, also presented similar expression patterns across the tissues, but these clusters did not cover all members in each PC. PC1 and PC5 showed high expression in all four tissues; while PC13 was leaf and phloem specific and PC6 was expressed lowly, especially in xylem, which can be a clue to understand their biological functions due to lack of homologous genes of which biological functions were characterized. Expression profiles of each tissue exhibited that some clustered GATA genes from the same PC were same as those in the four tissues and the rest were not (Fig. 4b–e), showing that expression level of *Populus* GATA genes partially reflects their conserveness across the species.

### Domain types of *Populus* GATA genes

The DNA-binding motif of GATA TFs was classified into three types designated as type IV_a_ (CX_2_CX_17_CX_2_C), IV_b_ (CX_2_CX_18_CX_2_C), and IV_c_ (CX_2_CX_20_CX_2_C) among which Type IV_b_ and IV_c_ are common in plants^25, 40, 81, 89^. Additional types, including type IV_p_ (p indicates partial; mentioned in *Arabidopsis*^43^ and *G. max* GATA TF analyses^25^) and type IV_4_ (four amino acids between the first two cysteines, which seems to be functionally active^43^), were also found.

In *Populus* GATA TFs, type IV_b_ is the most abundant (272 of 389; 69.92%), and type IV_c_ is the second (102; 26.22%). Type IV_b_, a common type of DNA-binding motifs of plant GATA TFs, occupies the largest proportion in *Populus* GATA TFs and is found in subfamilies I, II, and IV and Type IV_c_, the second largest, is a common in subfamily III of *Populus* GATA TFs, which is similar to those of *A. thaliana*, *G. max*, *V. vinifera*, and *O. sativa*. Type IV_4_ is found only in eight *Populus* GATA TFs (2.06%) belonging to subfamily II. It was also identified in some species including *A. thaliana* and *G. max*, suggesting that this type was independently occurred during evolution by adding two amino acids only in the first two cysteines of subfamily II. In contrast, type IV_p_ is found in all subfamilies of *Populus* GATA TFs as well as those of *A. thaliana*, *G. max*, and *O. sativa*, suggesting that random modifications of GATA domains of all subfamilies have been occurred during evolution. Interestingly, type IV_p_ can be generated by alternative splicing forms: PtaaGATA36d is GATA TF displaying type IV_p_; while the rest four alternative splicing forms of PtaaGATA36 show type IV_c_ domain, which is similar to the cases of OsGATA8^81^ and AtGATA26. Type IV_p_ was also considered as an ancestral form of GATA zinc finger^40^, requiring more research of Type IV_p_ with additional GATA genes from many plant genomes.

### Amino acid patterns of GATA domains in seven *Populus* species and *A. thaliana*

**Figure 5:**
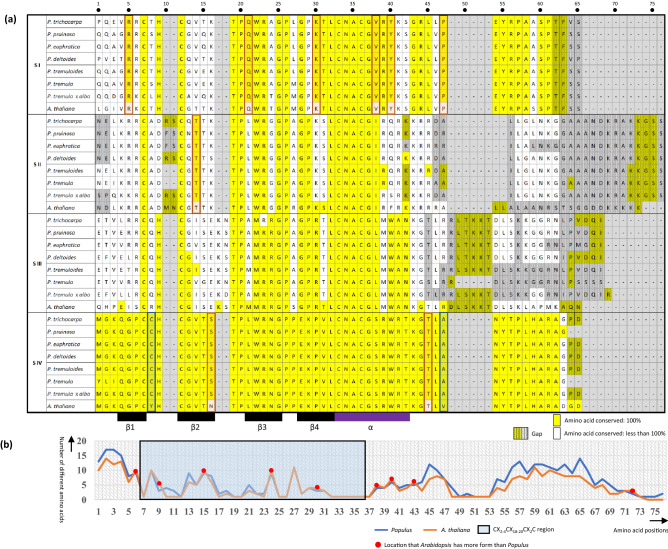
Conserved amino acids of GATA domain along with subfamilies and genera (*A. thaliana* and *Populus*). (**a**) shows the conserved amino acid in each position of the GATA domain. Yellow shaded characters mean 100% conserved amino acids and gray background color in amino acid indicate that there are gaps in the position. S I, S II, S III, and S IV are shortened forms of subfamily I, II, III, and IV, respectively. Blue-colored transparent boxes show incongruent of conserved amino acids between the two species. Red-colored transparent boxes present amino acids which are 100% conserved in the *Populus* genus but not in *A. thaliana*. (**b**) The X-axis indicates each amino acid position of the aligned amino acids of GATA domain and Y-axis displays the number of different amino acids in the specific position of aligned GATA domain of *Populus* (blue line) and *A. thaliana* (orange line). Red dots on the graph mean position where *A. thaliana* has more different amino acids than that of *Populus*. The blue-colored transparent box presents the CX_2_CX_18-20_CX_2_C region.

Amino acid sequences of 422 GATA domain from 382 *Populus* and 40 *A. thaliana* GATA TFs excluding seven type IV_p_ domains of *Populus* and one *Arabidopsis* were used for multiple sequence alignment (Fig. 5a). Subfamily IV of *Populus* displays the most conserved manner (49 of 55 conserved amino acids are identical.) in their domains, while subfamily II of *Populus* shows the least conserveness (18 of 66 conserved amino acids). Considering with the number of *Populus* GATA genes in each subfamily, subfamily I, the largest subfamily, is more conserved than subfamilies II and III. Some of dominant amino acids in GATA domain are different among *Populus* species (e.g., the 5’ region of GATA domains; Fig. 5a).

Subfamily IV displays incongruent of conserved amino acids between *Populus* and *A. thaliana* at 8th and 47th (Fig. 5a; blue-colored transparent boxes), indicating that subfamily IV was evolved and stabilized in early stage. In addition, six, one, and two amino acids which are 100% conserved in *Populus* genus but not in *A. thaliana* are found in subfamilies I, II, and IV, respectively (Fig. 5a; red-colored transparent boxes), suggesting that subfamily I has been most diversified in the lineage of *A. thaliana*.

Twelve conserved amino acids of *Populus* and *A. thaliana* belonging to the zinc finger motif (CX_2-4_CX_18-20_CX_2_C) are identical in all subfamilies (Fig. 5a) suggesting that the zinc finger motif is the most conserved and important region in the GATA domain. 22nd and 28th conserved amino acids in subfamilies I, II, and IV are Tryptophan and Glycine, respectively; while subfamily III displays methionine and glutamic acid (Fig. 5a). These differences can be key factors to classify four subfamilies.

As expected, the zinc finger motif, which can bind to DNA and is the most important region in GATA domain, contains a smaller number of different amino acids (Fig. 5b). Despite of large number of species in *Populus* genus used in this study, four positions in this region show a high number of amino acids in *A. thaliana* (Fig. 5b), suggesting that selection pressures have been differently applied in the two lineages. This phenomenon is also found outside this region (Fig. 5b). It is congruent to the findings described in the previous paragraph. Once more plant genomes including the large number of resequencing data of *A. thaliana* and *P. trichocarpa* are analyzed, the detailed evolutionary history of the GATA domain will be uncovered.

### Identification of transmembrane helix (TMH) of GATA gene family in seven *Populus* genomes

Membrane-bound transcription factors (MTFs) are docked in cellular membranes using their transmembrane domains^100^. MTFs have usually been found in plant species^101^ of which are related to seed germination^102^, cell division^101^, heat stress^103^, and salt stress^104^. Mechanisms of MTFs are well known in two major plant TF families: NAC TF family and bZIP TF family^105^ (Fig. S4). NTL6 (NAC TF) of *A. thaliana* is localized in the plasma membrane under normal conditions; while under stress conditions, NTL6 is processed by an as-yet-unidentified intramembrane protease and SnRK2.8 kinase phosphorylates NTL6 and facilitates its nuclear import^105^. (ii) Intracellular movement of *Arabidopsis* bZIP60 and bZIP28 was characterized^105^. bZIP60 and bZIP28, which are other MTFs in *Arabidopsis*, were localized on the membrane of the endoplasmic reticulum and then transported to nucleus by cleaving TMH.

Five *Populus* GATA TFs were identified with TMHs predicted by TMHMM^106^. PtrGATA14b, 14c (*P. trichocarpa*) in subfamily I, PpGATA21, 25 (*P. pruinosa*), and PtaaGATA23 (*P. tremula* x *alba*; Table S10), belonging to subfamily II. These putative GATA MTFs have one TMH, which is the same as the previously characterized *Arabidopsis* MTFs^107^. As far as we know, this is the first time to report putative GATA MTFs in plant species; additional putative GATA MTFs were also identified in other plant species using our pipeline: VvGATA19 (*V. vinifera*; subfamily IV)^89^, GmGATA39 (*G. max*; subfamily I)^40^, OsGATA14 (*O. sativa*; subfamily II)^81^ with one TMH, while no GATA MTF was found in *A. thaliana*^25^. It is interesting that there are no common subfamilies containing putative GATA MTFs along with different species. In addition, some alternative splicing forms of PtrGATA14 (*P. trichocarpa*) have TMH, indicating that truncation of TMH region by alternative splicing may switch their functions by changing subcellular localization of GATA TFs, similar to the bZIP60 in *A. thaliana*^105^. With accumulating more data including expression profiles and subcellular localization, roles of these putative GATA MTFs can be uncovered. Moreover, these results can be a corner stone to understand plant GATA MTFs together with a large number of plant genomes available now^108–110^ in a broad taxonomic range of plant species.

### Chromosomal distribution of *P. trichocarpa*,* P. tremula* x *alba*, and *P. deltoides* GATA gene family

**Figure 6:**
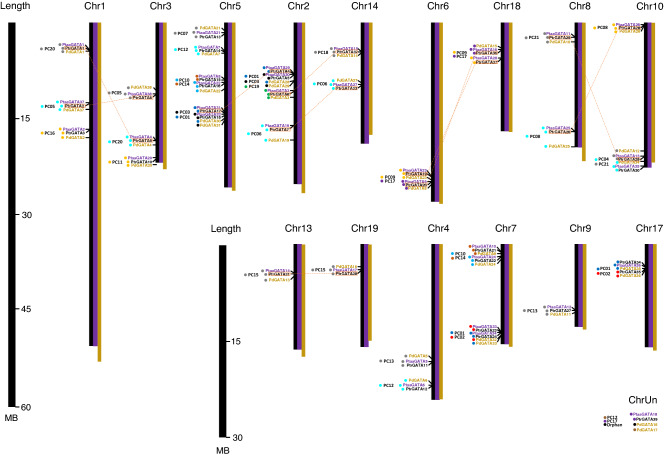
Chromosomal distribution of *P. trichocarpa*, *P. tremula* x *alba*, *and P. deltoides* GATA genes. Black, purple, and yellow bars indicate *P. trichocarpa*, *P. tremula* x *alba*, and *P. deltoides* chromosomes, respectively. Black, purple, yellow letters mean GATA gene names of *P. trichocarpa*, *P. tremula* x *alba*, and *P. deltoides*. ChrUn present scaffold sequences which are not assigned to any chromosomes. Yellow-colored transparent boxes indicate 10 paralogous pairs.

Chromosomal distribution of *Populus* GATA genes from the three species, *P. trichocarpa*, *P. tremula* x *alba*, and *P. deltoides* belonging to the same clade (Fig. 6), presents several important features: (i) 38 of 39 GATA genes in *P. trichocarpa*, 37 out of 38 in *P. tremula* x *alba*, and 36 of 38 in *P. deltoides* were distributed on 15 of 19 chromosomes (Fig. 6), presenting similar chromosomal distribution among three species. (ii) Chromosome 5 in both species contains the largest number of GATA genes; while chromosomes 9, 13, and 19 in both species contain the smallest (Fig. 6). This biased chromosomal distribution was also found in many plant species including *A. thaliana*^25^, *V. vinifera*^89^, *G. max*^40^, *O. sativa*^81^. (iii) In the three species, chromosome 7 shows the highest density of GATA genes in both species; and chromosome 5 is a second rank. (iv) Most of GATA genes of three species are in the same PCs and in similar chromosomal position (Fig. 6) except the four genes of which chromosomal positions are not assigned (See ChrUn in Fig. 6). It indicates that there might be no chromosomal rearrangement events and biological functions of GATA genes may have similar functions among the three species. Interestingly, three of the four genes are additional copy of GATA TFs in PC12 and PC17, which is the result of independent gene gain events.

Based on paralogous GATA TFs of *P. trichocarpa* identified in the previous study^111^, 10 paralogous pairs were successfully mapped to GATA TFs from three *Populus* species (Fig. 6), displaying that all paralogous pairs contain three GATA TF from each species in the similar chromosomal positions, indicating that gene duplication events of the 10 paralogous pairs were occurred before speciation of three *Populus* species (see yellow star in Fig. 1a).

In addition, three PCs, PC14 (PtrGATA15 and PtaaGATA8 in chromosome 5), PC02 (PtrGATA35 and PdGATA36 in chromosome 17), and PC09 (PtrGATA37 and PtaaGATA28 in chromosome 18), have not complete set of *Populus* GATA TFs. PC14 and PC09 suggest the loss event of GATA genes in the lineage of *P. deltoides* (see the light blue line in Fig. 1). PC02 indicates another loss event occurred in the lineage of *P. tremuloides* (See green lines in Fig. 1). Taken together with the incongruency inferred from the PCA, GATA TFs were evolved with the events occurred in various lineages in *Populus* species, which is independent to their species evolution.

## Conclusion

Using the identification pipeline of GATA TFs in the GATA-TFDB, we successfully identified 262 GATA genes (389 GATA TFs) from seven *Populus* species. Alternative splicing forms of *Populus* GATA genes display the high number of alternative splicing forms (nine is maximum) with only changes in untranslated regions and loss of DNA-binding motif or additional domains. *Populus* GATA genes were classified into the four subfamilies, same to *Arabidopsis* GATA genes, except that some genes in subfamily III lack CCT and/or TIFY domains. 21 *Populus* GATA gene clusters (PCs) were identified from the phylogenetic tree of GATA domain sequences and 20 of 21 PCs cover the seven *Populus* species, displaying the conserveness of *Populus* GATA genes. Distribution of alternative splicing forms in the PCs exhibits the possibility of subfunctionalization and neofunctionalization of *Populus* GATA genes. Through the expression analysis of GATA genes of two *Populus* species, the five PCs which display similar expression patterns across the four tissues were identified for predicting their biological functions. *Populus*-specific conserved amino acids in the GATA domain were discovered in comparison to *A. thaliana*, suggesting a complex evolutionary history of the GATA domain. Five *Populus* GATA TFs contain one transmembrane helix (TMH), which is the first report of membrane-bound GATA TFs. Together with the biased distribution of GATA genes across the chromosomes, paralogous pairs of GATA genes suggested several gene duplication events in the lineages of *Populus* genus. Taken together, our first comprehensive analyses of genus-wide GATA TFs in plants successfully provide characteristics of *Populus* GATA genes across the seven species as well as their putative functions and evolutionary traits of *Populus* GATA genes.

## Materials and methods

### Collecting *Populus* genome sequences from various sources

We utilized the seven *Populus* genomes sequences deposited from the *Populus* Comparative Genome Database^108–110^ (http://www.populusgenome.info/), which adopted-whole genome sequences from the Plant Genome Database (http://www.plantgenome.info/; Park et al., in preparation). These genomes were originated from the NCBI genome database (http://genome.ncbi.nlm.nih.gov/) and Phytozome (http://www.phyotozome.info/)^112^ in the standardized form of genome sequences provided by the GenomeArchive® (http://www.genomearchive.info/)^113^.

### Identifying GATA TFs from whole *Populus* genome sequences

Amino acid sequences from seven *Populus* genomes were subjected to InterProScan^114^ to identify GATA TFs. The pipeline for identifying *Populus* GATA TFs implemented at the GATA-TFDB (http://gata.genefamily.info/; Park et al., in preparation), which is an automated pipeline for identifying GATA TFs with GATA DNA-binding motif InterPro term (IPR000679) and post-process to filter-out false positive results and for analyzing various analyses including domain sequence analysis, gene family analysis, as well as phylogenetic analysis. GATA-TFDB was constructed and maintained as one of members of the Gene Family Database (http://www.genefamily.info/; Park et al., in preparation).

### Exon structure and alternative splicing forms of *Populus* GATA TFs

Based on the *Populus* Comparative Genome Database (http://www.populusgenome.info/; Park et al., in preparation), exon structure and alternative splicing forms of GATA TFs were retrieved. Diagrams of exon structure and alternative splicing forms of GATA TFs were drawn primarily based on the diagram generated by the GATA-TFDB (http://gata.genefamily.info; Park et al., in preparation) with adding additional information manually.

### Construction of phylogenetic tree of *Populus* GATA TFs

Phylogenetic trees were constructed with a Neighbor-joining method with bootstrap option (10,000 repeats) by ClustalW 2.1^115^ based on the alignment of amino acids of GATA domains obtained from the GATA-TFDB (http://gata.genefamily.info; Park et al., in preparation) also by ClustalW 2.1^115^.

### Chromosomal distribution of *Populus* GATA TFs

We drew the chromosomal distribution map of *Populus* GATA genes from three species based on the chromosomal coordination from their pseudo-molecule level assemblies deposited in the Plant Genome Database (http://www.plantgenome.info/).

### Prediction of transmembrane helixes on *Populus* GATA TFs

Transmembrane helixes on *Populus* GATA TFs were predicted by TMHMM^106^ under the environment of the Plant Genome Database (http://www.plantgenome.info/).

### Principal component analysis of *Populus* GATA TFs

Principal component analysis (PCA) was conducted based on 19 characteristics of GATA genes using *prcomp* function in R package stats version 4.0.3^116^. The result was visualized into a scatterplot including variance, with the first two principal components.

### Expression analysis of GATA TFs based on *Populus* RNA-seq data

Raw reads of RNA-Seq experiments of *P. pruinosa* and *P. deltoides* were downloaded from NCBI (Table S9). RNA-Seq raw reads were aligned against the whole genome of *P. pruinosa* and *P. deltoides* with hisat2 v2.2.0^117^ after generating datasets of each *Populus* genome. After generating bam file for each SRA raw reads, bam files from the same experiments were merged using samtools v1.9^118^. Expression levels of the merged bam files were calculated by cufflink v2.2.1^119^.

Hierarchical clustering was conducted for the five datasets: one covers four different conditions (four tissues) and the last four contain each condition (Fig. 4) using *hclust* function in R package stats version 4.0.3^116^.

### Construction of phylogenetic tree of seven *Populus* species based on complete chloroplast genomes

Complete chloroplast genomes of seven *Populus* chloroplast genomes^69, 120–123^ and *Salix gracilistyla*^78^, used as an outgroup, were aligned using MAFFT v7.450^124^. All chloroplast genome sequences were retrieved from the PCD (http://www.cp-genome.net; Park et al., in preparation). The maximum-likelihood trees were reconstructed in MEGA X^125^. During the ML analysis, a heuristic search was used with nearest-neighbor interchange branch swapping, the Tamura-Nei model, and uniform rates among sites. All other options were set to their default values. Bootstrap analyses with 1,000 pseudoreplicates were conducted with the same options. All bioinformatic processes were conducted under the environment of the Genome Information System (GeIS) used in the various previous studies^43, 126–134^.

## Supplementary Information

Supplementary Information 1.

Supplementary Information 2.

Supplementary Information 3.

Supplementary Information 4.

Supplementary Information 5.

Supplementary Information 6.

Supplementary Information 7.

Supplementary Information 8.

Supplementary Information 9.

Supplementary Information 10.

Supplementary Information 11.

Supplementary Information 12.

Supplementary Information 13.

Supplementary Information 14.

Supplementary Information 15.

## Data Availability

All GATA TFs identified in this study can be accessed at the *Populus* Comparative Genome Database (http://www.populusgenome.info/).
