# Supplementary figures and images for: Genome-wide comparative analyses of GATA transcription factors among seven Populus genomes
Source: Sci Rep. 2021 Aug 16;11:16578. doi: 10.1038/s41598-021-95940-5 (PMC8367991; doi:10.1038/s41598-021-95940-5)

## Slide 1
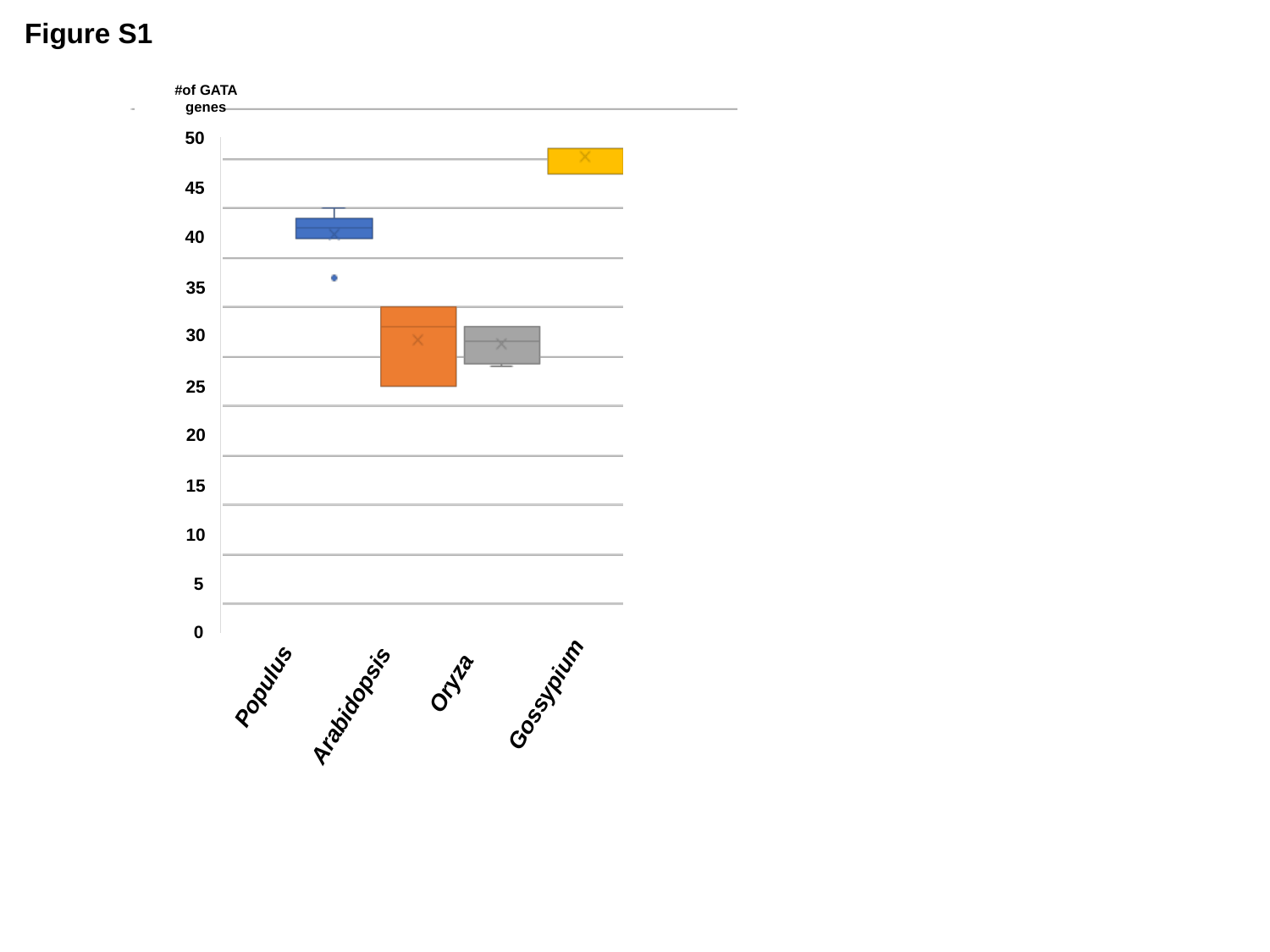

Figure S1
#of GATA genes
50
45
40
35
30
25
20
15
10
5
0
Oryza
Populus
Gossypium
Arabidopsis

Supplement: Supplementary file 1 — Supplementary Information 1. [file 41598_2021_95940_MOESM1_ESM.pptx]

## Slide 1
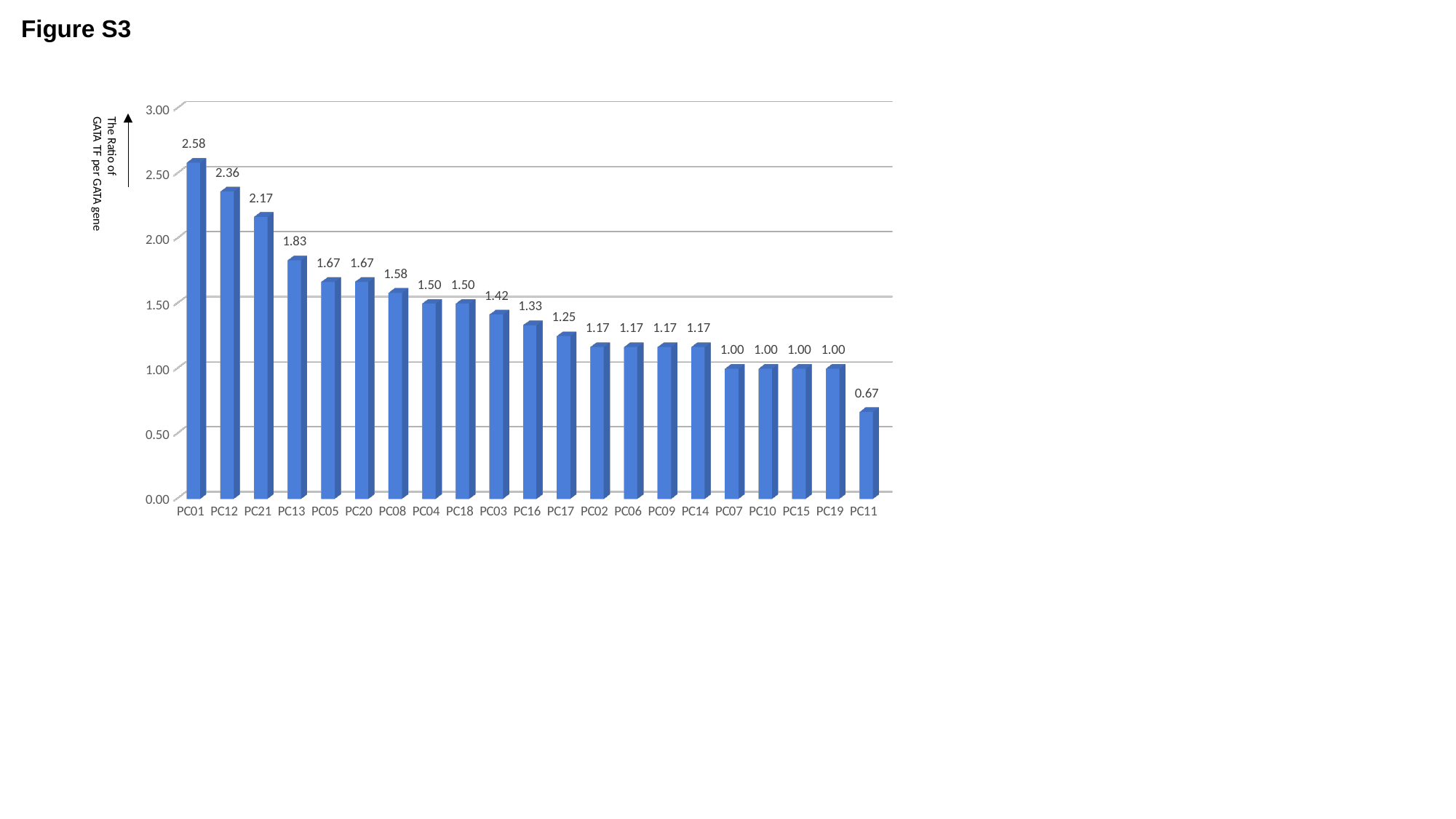

Figure S3
[unsupported chart]
The Ratio of
GATA TF per GATA gene

Supplement: Supplementary file 3 — Supplementary Information 3. [file 41598_2021_95940_MOESM3_ESM.pptx]
