# Supplementary material for: Genome-wide comparative analyses of GATA transcription factors among seven Populus genomes
Source: Sci Rep. 2021 Aug 16;11:16578. doi: 10.1038/s41598-021-95940-5 (PMC8367991; doi:10.1038/s41598-021-95940-5)
Supplement: Supplementary file 2 — Supplementary Information 2. [file 41598_2021_95940_MOESM2_ESM.pptx]

## Slide 1
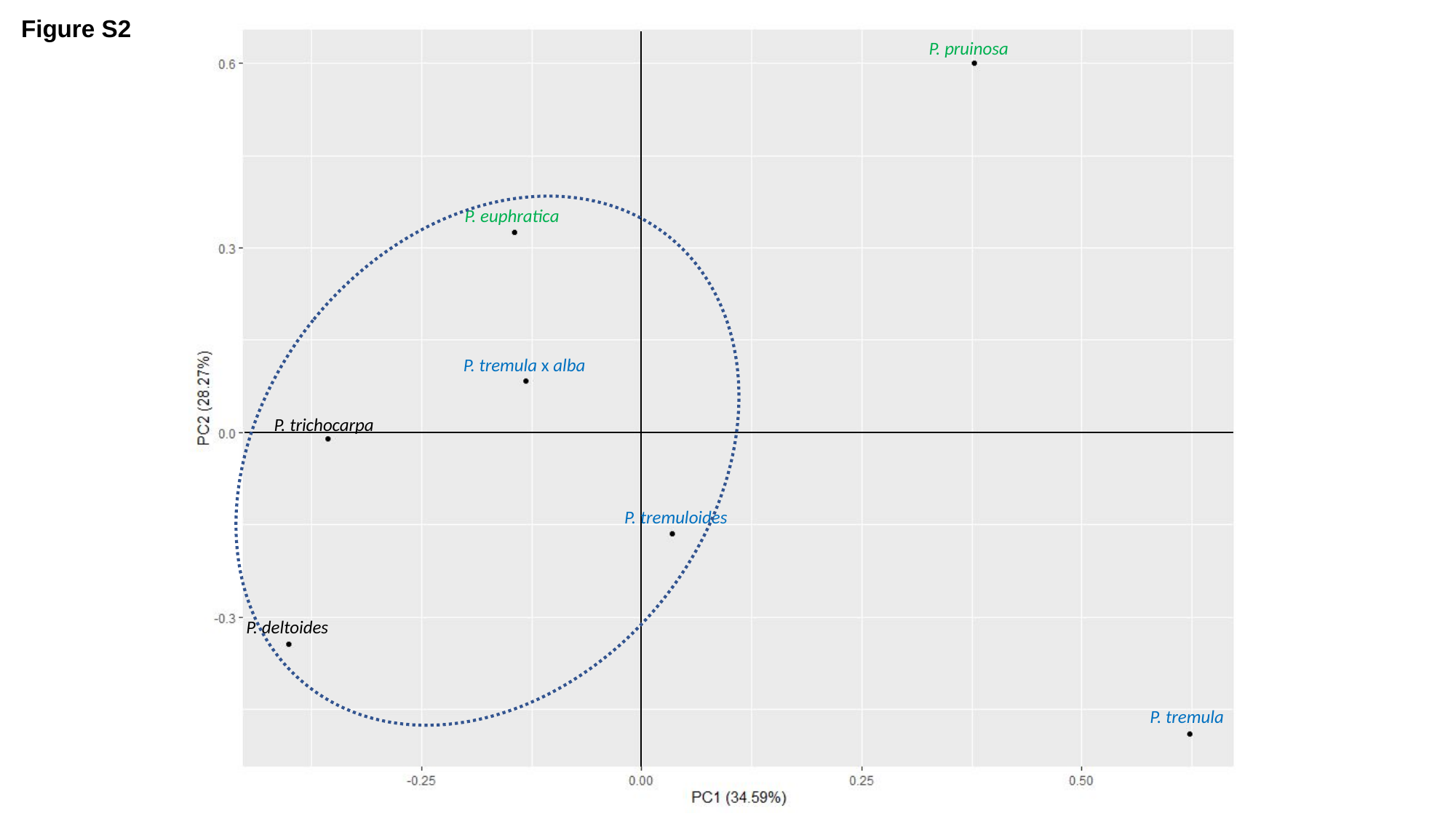

Figure S2
P. pruinosa
P. euphratica
P. tremula x alba
P. trichocarpa
P. tremuloides
P. deltoides
P. tremula
