# Supplementary material for: Genome-wide comparative analyses of GATA transcription factors among seven Populus genomes
Source: Sci Rep. 2021 Aug 16;11:16578. doi: 10.1038/s41598-021-95940-5 (PMC8367991; doi:10.1038/s41598-021-95940-5)
Supplement: Supplementary file 4 — Supplementary Information 4. [file 41598_2021_95940_MOESM4_ESM.pptx]

## Slide 1
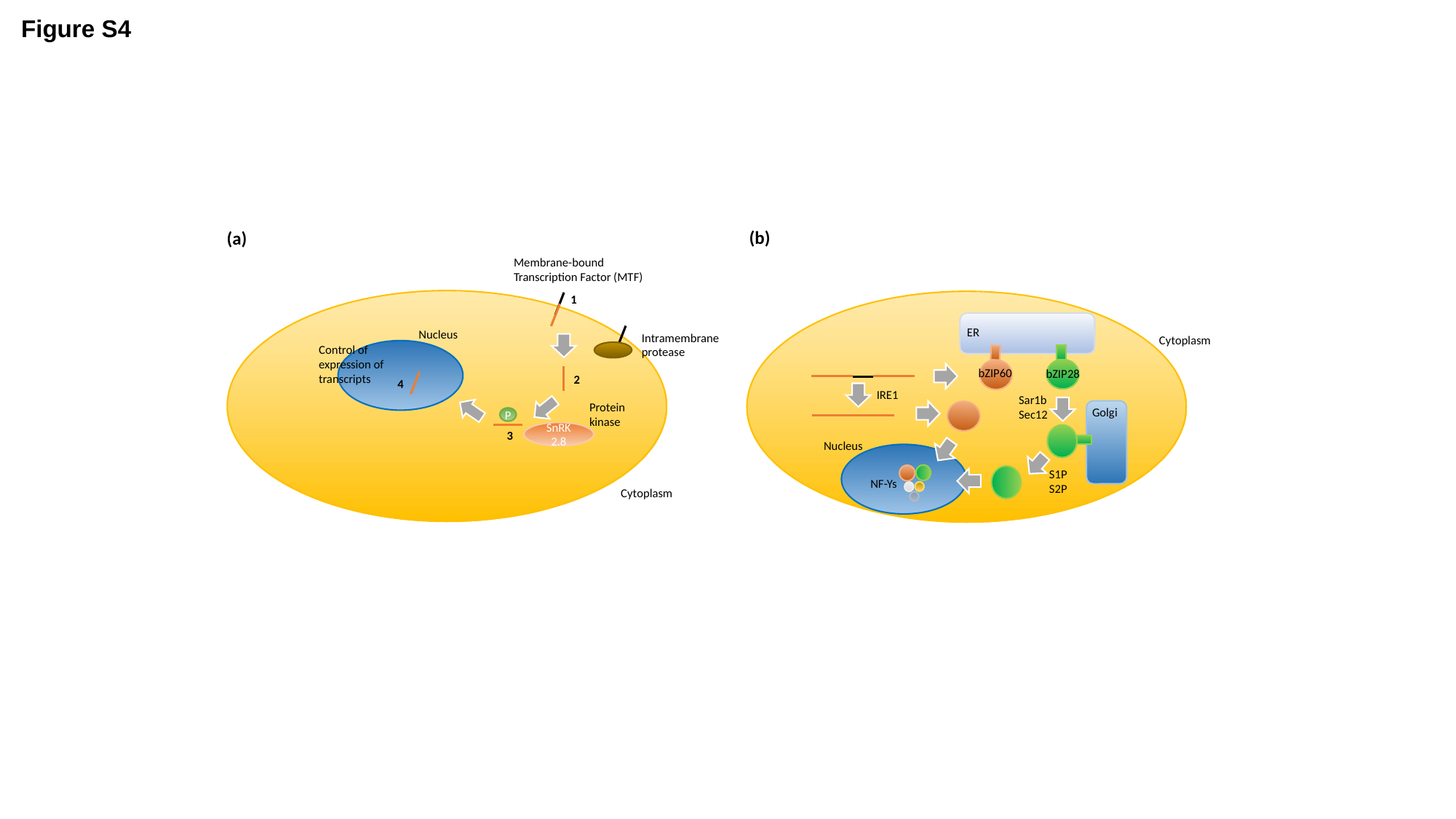

Figure S4
(b)
(a)
Membrane-bound Transcription Factor (MTF)
1
Nucleus
Intramembrane protease
Control of expression of transcripts
2
4
Protein kinase
P
SnRK
2.8
3
Cytoplasm
ER
Cytoplasm
bZIP60
bZIP28
IRE1
Sar1b
Sec12
Golgi
Nucleus
S1P
S2P
NF-Ys
