# Supplementary material for: Genome-wide comparative analyses of GATA transcription factors among seven Populus genomes
Source: Sci Rep. 2021 Aug 16;11:16578. doi: 10.1038/s41598-021-95940-5 (PMC8367991; doi:10.1038/s41598-021-95940-5)
Supplement: Supplementary file 5 — Supplementary Information 5. [file 41598_2021_95940_MOESM5_ESM.docx]

**Supplementary figure and table legends**

**Table S1.** Number of GATA genes in each subfamily of plant species used in genome-wide identification of GATA gene family (DOCX)

**Table S2.** List of identified 389 GATA TFs from 7 *Populus* genomes (DOCX)

**Table S3.** Types and positions of alternative splicing forms of GATA genes in six *Populus* (XLSX)

**Table S4.** RNA-Seq data of *Populus* genus deposited in NCBI (DOCX)

**Table S5.** Number of *Populus* GATA TFs without the GATA domain (DOCX)

**Table S6.** Number of *Populus* GATA genes in each subfamily (DOCX)

**Table S7.** List of identified GATA TFs of 21 *Populus* GATA gene clusters (PCs) (DOCX)

**Table S8.** Characteristics of 21 *Populus* GATA gene clusters (PCs) (DOCX)

**Table S9.** List of RNA-Seq raw reads used for analyzing expression level of GATA TFs (DOCX)

**Table S10.** List of GATA TFs having TMHs predicted by TMHMM 2.0 (DOCX)

**Figure S1. Numbers of GATA genes in the four plant genera.** The X-axis indicates four plant genera, *Populus*, *Arabidopsis*, *Oryza*, and *Gossypium*, and Y-axis displays the number of GATA genes with a mean (gray-colored central cross) and median (gray central line).

**Figure S2. Principal component analysis of 19 GATA characteristics of among 7 *Populus*.** It presents the two-dimensional model among 7 *Populus* originated from principal component analysis of 19 characteristics of *Populus* GATA genes. A circle with a blue dotted line means a *Populus* group.

**Figure S3. The ratio of the number of GATA TF per GATA gene in 21 PCs.**

**Figure S4. Mechanisms of two membrane-bound TFs, (a) NAC TF (NTL6) and (b) bZIP TFs (bZIP28 and bZIP60).** This diagram was re-drawn based on the publication of MTFs^105^.
