# Supplementary material for: Genome-wide comparative analyses of GATA transcription factors among seven Populus genomes
Source: Sci Rep. 2021 Aug 16;11:16578. doi: 10.1038/s41598-021-95940-5 (PMC8367991; doi:10.1038/s41598-021-95940-5)
Supplement: Supplementary file 6 — Supplementary Information 6. [file 41598_2021_95940_MOESM6_ESM.docx]

**Table S1.** Number of GATA genes in each subfamily of plant species used in genome-wide identification of GATA gene family

| **Plant genome names** | **Number of each subfamily of GATA genes** | | | | | | | **Total** | **Ref.** |
| --- | --- | --- | --- | --- | --- | --- | --- | --- | --- |
|  | **I** | **II** | **III** | **IV** | **V** | **VI** | **VII** |  |  |
| *Arabidopsis thaliana* | 14 | 11^*^ | 3 | 2 | 0 | 0 | 0 | 30 | ^1^ |
| *Glycine max* | 30 | 17 | 9 | 8 | 0 | 0 | 0 | 64 | ^2^ |
| *Gossypium arboreum* | 20 | 13 | 8 | 5 | 0 | 0 | 0 | 46 | ^3^ |
| *Gossypium hirsutum* | 36 | 25 | 16 | 10 | 0 | 0 | 0 | 87 | ^3^ |
| *Gossypium raimondii* | 19 | 14 | 8 | 5 | 0 | 0 | 0 | 46 | ^3^ |
| *Malus domestica* | 20 | 8 | 4 | 3 | 0 | 0 | 0 | 35 | ^4^ |
| *Ricinus communis* | 7 | 7 | 4 | 1 | 0 | 0 | 0 | 19 | ^5^ |
| *Solanum lycopersicum* | 14 | 9 | 4 | 3 | 0 | 0 | 0 | 30 | ^6^ |
| *Populus trichocarpa* | 18 | 10 | 9 | 2 | 0 | 0 | 0 | 39 | ^7^ |
| *Brassica napus* | 36 | 43 | 10 | 7 | 0 | 0 | 0 | 96 | ^8^ |
| *Ophiorrhiza pumila* | 7 | 5 | 5 | 1 | 0 | 0 | 0 | 18 | ^9^ |
| *Vitis vinifera* | 7 | 6 | 5 | 1 | 0 | 0 | 0 | 19 | ^10^ |
| *Oryza sativa* | 7 | 9 | 5 | 0 | 2 | 3 | 2 | 28 | ^1^ |
| *Triticum aestivum* | 13 | 6 | 4 | 3 | 0 | 0 | 0 | 26 | ^11^ |
| *Phyllostachys edulis* | 12 | 13 | 6 | 0 | 0 | 0 | 0 | 31 | ^12^ |
| **Total** | **260** | **196** | **100** | **51** | **2** | **3** | **2** | **614** |  |

^*^This number is from the recent analysis^13^.

^**^*Piper nigrum*, *Zea mays*, *Solanum tuberosum*, and *Capsicum annuum* were omitted because their paper could not be accessed^14-17^.

^***^In the case of two species, different classification, group A, B, C, and/or D, was used so that it is also omitted (group A: 15 GATA genes, group B: 5 GATA genes, group C: 7 GATA genes, and group D: 1 GATA genes in *Brachypodium distachyon*^18^ and group A: 17 GATA genes, group B: 5 GATA genes, and group C: 3 GATA genes in *Cicer arietinum*^19^).

1. Reyes, J. C., Muro-Pastor, M. I. & Florencio, F. J. The GATA family of transcription factors in *Arabidopsis* and rice. *Plant physiology* **134**, 1718-1732 (2004).

2. Zhang, C. *et al.* Genome-wide survey of the soybean GATA transcription factor gene family and expression analysis under low nitrogen stress. *PLoS One* **10**, e0125174 (2015).

3. Zhang, Z. *et al.* Genome-wide identification and analysis of the evolution and expression patterns of the GATA transcription factors in three species of *Gossypium* genus. *Gene* (2018).

4. Chen, H. *et al.* Genome-wide identification, evolution, and expression analysis of GATA transcription factors in apple (*Malus× domestica* Borkh.). *Gene* **627**, 460-472 (2017).

5. Ao, T., Liao, X., Xu, W. & Liu, A. Identification and characterization of GATA gene family in Castor Bean (*Ricinus communis*). *Plant Diver. Resour.* **37**, 453-462 (2015).

6. Yuan, Q., Zhang, C., Zhao, T., Yao, M. & Xu, X. A Genome-Wide Analysis of GATA Transcription Factor Family in Tomato and Analysis of Expression Patterns. *INTERNATIONAL JOURNAL OF AGRICULTURE BIOLOGY* **20**, 1274-1282 (2018).

7. Apuli, R.-P. *et al.* Inferring the genomic landscape of recombination rate variation in European aspen (Populus tremula). *G3: Genes, Genomes, Genetics* **10**, 299-309 (2020).

8. Zhu, W., Guo, Y., Chen, Y., Wu, D. & Jiang, L. Genome-Wide Identification and Characterization of GATA Family Genes in *Brassica Napus*. (2020).

9. Huang, Q., Shi, M., Wang, C., Hu, J. & Kai, G. Genome-wide Survey of the GATA Gene Family in Camptothecin-producing Plant Ophiorrhiza Pumila. (2021).

10. Zhang, Z. *et al.* Characterization of the GATA gene family in *Vitis vinifera*: genome-wide analysis, expression profiles, and involvement in light and phytohormone response. *Genome* **61**, 713-723 (2018).

11. Liu, H. *et al.* TaZIM‐A1 negatively regulates flowering time in common wheat (*Triticum aestivum* L.). *Journal of integrative plant biology* (2018).

12. Wang, T. *et al.* Genome-wide analysis of GATA factors in moso bamboo (Phyllostachys edulis) unveils that PeGATAs regulate shoot rapid-growth and rhizome development. *bioRxiv*, 744003 (2019).

13. Kim, M., Xi, H. & Park, J. Genome-wide comparative analyses of GATA transcription factors among 19 Arabidopsis ecotype genomes: Intraspecific characteristics of GATA transcription factors. *PloS one* **16**, e0252181 (2021).

14. Qi, Y., Chunli, Z., Tingting, Z. & Xiangyang, X. Bioinformatics Analysis of GATA Transcription Factor in Pepper. *Chinese Agricultural Science Bulletin* **2017**, 5 (2017).

15. Jiang, L., Yu, X., Chen, D., Feng, H. & Li, J. Identification, phylogenetic evolution and expression analysis of GATA transcription factor family in maize (*Zea mays*). *International Journal of Agriculture and Biology* **23**, 637-643 (2020).

16. Yu, R. *et al.* Genome-wide identification of the GATA gene family in potato (Solanum tuberosum L.) and expression analysis. *Journal of Plant Biochemistry and Biotechnology*, 1-12 (2021).

17. Yu, C. *et al.* Genome-wide identification and function characterization of GATA transcription factors during development and in response to abiotic stresses and hormone treatments in pepper. *Journal of Applied Genetics* **62**, 265-280 (2021).

18. Peng, W. *et al.* Genome-Wide Characterization, Evolution, and Expression Profile Analysis of GATA Transcription Factors in Brachypodium distachyon. *International journal of molecular sciences* **22**, 2026 (2021).

19. Niu, L. *et al.* The GATA gene family in chickpea: structure analysis and transcriptional responses to abscisic acid and dehydration treatments revealed potential genes involved in drought adaptation. *Journal of Plant Growth Regulation* **39**, 1647-1660 (2020).
