# Supplementary material for: Genome-wide comparative analyses of GATA transcription factors among seven Populus genomes
Source: Sci Rep. 2021 Aug 16;11:16578. doi: 10.1038/s41598-021-95940-5 (PMC8367991; doi:10.1038/s41598-021-95940-5)
Supplement: Supplementary file 7 — Supplementary Information 7. [file 41598_2021_95940_MOESM7_ESM.docx]

**Table S2.** List of identified 389 GATA TFs from seven *Populus* genomes

| **No** | **Species** | **Sub-family** | **GATA name** | **Protein name** | **Length (aa)** | **# of exons** | **Chro- mosome** | **Genomic coordination** |
| --- | --- | --- | --- | --- | --- | --- | --- | --- |
| 1 | *P. trichocarpa* | I | PtrGATA1 | Potri.001G053500.1.v3.1 | 336 | 2 | Chr01 | 4087126-4088760 |
| 2 | *P. trichocarpa* | IV | PtrGATA2a | Potri.001G151700.2.v3.1 | 544 | 8 | Chr01 | 12503731-12509077 |
| 3 | *P. trichocarpa* | IV | PtrGATA2b | Potri.001G151700.4.v3.1 | 552 | 9 | Chr01 | 12503731-12509374 |
| 4 | *P. trichocarpa* | IV | PtrGATA2c | Potri.001G151700.5.v3.1 | 550 | 9 | Chr01 | 12503731-12509260 |
| 5 | *P. trichocarpa* | I | PtrGATA3 | Potri.001G188500.2.v3.1 | 200 | 1 | Chr01 | 16658793-16659395 |
| 6 | *P. trichocarpa* | III | PtrGATA4a | Potri.002G110800.1.v3.1 | 360 | 10 | Chr02 | 8198641-8203370 |
| 7 | *P. trichocarpa* | III | PtrGATA4b | Potri.002G110800.5.v3.1 | 338 | 9 | Chr02 | 8199477-8203370 |
| 8 | *P. trichocarpa* | III | PtrGATA5 | Potri.002G110900.1.v3.1 | 290 | 7 | Chr02 | 8206778-8211458 |
| 9 | *P. trichocarpa* | I | PtrGATA6 | Potri.002G142800.1.v3.1 | 246 | 2 | Chr02 | 10597705-10598590 |
| 10 | *P. trichocarpa* | II | PtrGATA7 | Potri.002G199800.1.v3.1 | 147 | 3 | Chr02 | 16060548-16061209 |
| 11 | *P. trichocarpa* | IV | PtrGATA8a | Potri.003G082800.1.v3.1 | 540 | 8 | Chr03 | 11054498-11059957 |
| 12 | *P. trichocarpa* | IV | PtrGATA8b | Potri.003G082800.2.v3.1 | 527 | 8 | Chr03 | 11054498-11059957 |
| 13 | *P. trichocarpa* | I | PtrGATA9 | Potri.003G174800.2.v3.1 | 258 | 2 | Chr03 | 18351510-18352918 |
| 14 | *P. trichocarpa* | II | PtrGATA10 | Potri.003G213300.1.v3.1 | 226 | 3 | Chr03 | 21117706-21119661 |
| 15 | *P. trichocarpa* | I | PtrGATA11a | Potri.004G161500.1.v3.1 | 327 | 2 | Chr04 | 18222377-18223450 |
| 16 | *P. trichocarpa* | I | PtrGATA11b | Potri.004G161500.2.v3.1 | 327 | 2 | Chr04 | 18222377-18223450 |
| 17 | *P. trichocarpa* | I | PtrGATA12a | Potri.004G211800.1.v3.1 | 301 | 3 | Chr04 | 21952453-21955852 |
| 18 | *P. trichocarpa* | I | PtrGATA12b | Potri.004G211800.2.v3.1 | 301 | 3 | Chr04 | 21952453-21955852 |
| 19 | *P. trichocarpa* | I | PtrGATA12c | Potri.004G211800.3.v3.1 | 301 | 3 | Chr04 | 21952453-21955852 |
| 20 | *P. trichocarpa* | I | PtrGATA12d | Potri.004G211800.4.v3.1 | 295 | 3 | Chr04 | 21952453-21955852 |
| 21 | *P. trichocarpa* | I | PtrGATA12e | Potri.004G211800.5.v3.1 | 295 | 3 | Chr04 | 21952453-21955852 |
| 22 | *P. trichocarpa* | I | PtrGATA12f | Potri.004G211800.6.v3.1 | 235 | 2 | Chr04 | 21952453-21954946 |
| 23 | *P. trichocarpa* | I | PtrGATA12g | Potri.004G211800.7.v3.1 | 235 | 2 | Chr04 | 21952453-21954946 |
| 24 | *P. trichocarpa* | II | PtrGATA13 | Potri.005G020500.1.v3.1 | 161 | 3 | Chr05 | 1593756-1594912 |
| 25 | *P. trichocarpa* | I | PtrGATA14a | Potri.005G066100.2.v3.1 | 240 | 3 | Chr05 | 4766766-4769647 |
| 26 | *P. trichocarpa* | I | PtrGATA14b | Potri.005G066100.3.v3.1 | 236 | 1 | Chr05 | 4766766-4767476 |
| 27 | *P. trichocarpa* | I | PtrGATA14c | Potri.005G066100.4.v3.1 | 236 | 1 | Chr05 | 4766766-4767476 |
| 28 | *P. trichocarpa* | I | PtrGATA14d | Potri.005G066100.5.v3.1 | 188 | 2 | Chr05 | 4766766-4769278 |
| 29 | *P. trichocarpa* | I | PtrGATA14e | Potri.005G066100.6.v3.1 | 188 | 2 | Chr05 | 4766766-4769278 |
| 30 | *P. trichocarpa* | I | PtrGATA15 | Potri.005G117600.1.v3.1 | 333 | 2 | Chr05 | 9096795-9097892 |
| 31 | *P. trichocarpa* | II | PtrGATA16 | Potri.005G122700.1.v3.1 | 254 | 2 | Chr05 | 9549918-9550786 |
| 32 | *P. trichocarpa* | III | PtrGATA17a | Potri.005G152500.1.v3.1 | 365 | 10 | Chr05 | 14348550-14353120 |
| 33 | *P. trichocarpa* | III | PtrGATA17b | Potri.005G152500.3.v3.1 | 324 | 8 | Chr05 | 14349509-14353120 |
| 34 | *P. trichocarpa* | III | PtrGATA17c | Potri.005G152500.6.v3.1 | 321 | 9 | Chr05 | 14349258-14353120 |
| 35 | *P. trichocarpa* | III | PtrGATA18 | Potri.005G152800.1.v3.1 | 288 | 7 | Chr05 | 14386768-14398559 |
| 36 | *P. trichocarpa* | II | PtrGATA19 | Potri.006G229200.3.v3.1 | 303 | 3 | Chr06 | 24013241-24014681 |
| 37 | *P. trichocarpa* | I | PtrGATA20 | Potri.006G237700.1.v3.1 | 373 | 2 | Chr06 | 24697617-24698877 |
| 38 | *P. trichocarpa* | I | PtrGATA21 | Potri.007G016600.1.v3.1 | 376 | 3 | Chr07 | 1264247-1265600 |
| 39 | *P. trichocarpa* | II | PtrGATA22 | Potri.007G024500.1.v3.1 | 254 | 2 | Chr07 | 1850016-1850914 |
| 40 | *P. trichocarpa* | III | PtrGATA23 | Potri.007G116600.1.v3.1 | 142 | 4 | Chr07 | 13732114-13733395 |
| 41 | *P. trichocarpa* | III | PtrGATA24a | Potri.007G116700.1.v3.1 | 384 | 11 | Chr07 | 13736463-13741856 |
| 42 | *P. trichocarpa* | III | PtrGATA24b | Potri.007G116700.2.v3.1 | 362 | 10 | Chr07 | 13736463-13741856 |
| 43 | *P. trichocarpa* | I | PtrGATA25a | Potri.008G038900.1.v3.1 | 354 | 2 | Chr08 | 2195942-2197684 |
| 44 | *P. trichocarpa* | I | PtrGATA25b | Potri.008G038900.2.v3.1 | 354 | 2 | Chr08 | 2195942-2197684 |
| 45 | *P. trichocarpa* | I | PtrGATA25c | Potri.008G038900.3.v3.1 | 354 | 2 | Chr08 | 2195942-2197684 |
| 46 | *P. trichocarpa* | II | PtrGATA26 | Potri.008G213900.1.v3.1 | 138 | 3 | Chr08 | 16937785-16938400 |
| 47 | *P. trichocarpa* | I | PtrGATA27a | Potri.009G123400.1.v3.1 | 329 | 2 | Chr09 | 10283615-10284696 |
| 48 | *P. trichocarpa* | I | PtrGATA27b | Potri.009G123400.2.v3.1 | 329 | 2 | Chr09 | 10283615-10284696 |
| 49 | *P. trichocarpa* | I | PtrGATA27c | Potri.009G123400.3.v3.1 | 329 | 2 | Chr09 | 10283615-10284696 |
| 50 | *P. trichocarpa* | II | PtrGATA28a | Potri.010G001300.1.v3.1 | 153 | 2 | Chr10 | 150174-150731 |
| 51 | *P. trichocarpa* | II | PtrGATA28b | Potri.010G001300.2.v3.1 | 149 | 3 | Chr10 | 150174-150813 |
| 52 | *P. trichocarpa* | II | PtrGATA28c | Potri.010G001300.3.v3.1 | 148 | 3 | Chr10 | 150174-150813 |
| 53 | *P. trichocarpa* | I | PtrGATA29a | Potri.010G223300.1.v3.1 | 446 | 4 | Chr10 | 20747924-20750787 |
| 54 | *P. trichocarpa* | I | PtrGATA29b | Potri.010G223300.2.v3.1 | 352 | 2 | Chr10 | 20747924-20749584 |
| 55 | *P. trichocarpa* | I | PtrGATA29c | Potri.010G223300.3.v3.1 | 352 | 2 | Chr10 | 20747924-20749584 |
| 56 | *P. trichocarpa* | III | PtrGATA30 | Potri.010G251600.1.v3.1 | 307 | 7 | Chr10 | 22342358-22345183 |
| 57 | *P. trichocarpa* | I | PtrGATA31 | Potri.013G059600.1.v3.1 | 295 | 2 | Chr13 | 4460724-4462025 |
| 58 | *P. trichocarpa* | I | PtrGATA32 | Potri.014G058600.1.v3.1 | 251 | 2 | Chr14 | 4540385-4541240 |
| 59 | *P. trichocarpa* | II | PtrGATA33 | Potri.014G124400.1.v3.1 | 133 | 3 | Chr14 | 9595392-9595995 |
| 60 | *P. trichocarpa* | III | PtrGATA34a | Potri.017G042200.2.v3.1 | 383 | 11 | Chr17 | 3570740-3576635 |
| 61 | *P. trichocarpa* | III | PtrGATA34b | Potri.017G042200.3.v3.1 | 361 | 10 | Chr17 | 3570740-3576635 |
| 62 | *P. trichocarpa* | III | PtrGATA34c | Potri.017G042200.6.v3.1 | 372 | 10 | Chr17 | 3570885-3576635 |
| 63 | *P. trichocarpa* | III | PtrGATA35 | Potri.017G042300.2.v3.1 | 100 | 1 | Chr17 | 3583599-3583901 |
| 64 | *P. trichocarpa* | I | PtrGATA36 | Potri.018G044900.1.v3.1 | 380 | 2 | Chr18 | 4079002-4080273 |
| 65 | *P. trichocarpa* | II | PtrGATA37 | Potri.018G053600.1.v3.1 | 303 | 3 | Chr18 | 5587125-5588558 |
| 66 | *P. trichocarpa* | I | PtrGATA38 | Potri.019G033000.1.v3.1 | 294 | 2 | Chr19 | 3789140-3790372 |
| 67 | *P. trichocarpa* | I | PtrGATA39 | Potri.T172039.1.v3.1 | 210 | 1 | scaffold_694 | 5281-5913 |
| 68 | *P. deltoides* | I | PdGATA1a | Podel.01G058800.1 | 270 | 3 | Chr01 | 4505049-4508450 |
| 69 | *P. deltoides* | I | PdGATA1b | Podel.01G058800.2 | 258 | 3 | Chr01 | 4505080-4508450 |
| 70 | *P. deltoides* | I | PdGATA1c | Podel.01G058800.3 | 258 | 2 | Chr01 | 4507056-4508450 |
| 71 | *P. deltoides* | I | PdGATA1d | Podel.01G058800.4 | 258 | 2 | Chr01 | 4507056-4508450 |
| 72 | *P. deltoides* | I | PdGATA1e | Podel.01G058800.5 | 258 | 3 | Chr01 | 4505080-4508450 |
| 73 | *P. deltoides* | I | PdGATA2 | Podel.01G199000.1 | 306 | 2 | Chr01 | 17591438-17592528 |
| 74 | *P. deltoides* | I | PdGATA3 | Podel.02G156500.1 | 246 | 2 | Chr02 | 11552022-11552910 |
| 75 | *P. deltoides* | I | PdGATA4 | Podel.03G187800.1 | 258 | 2 | Chr03 | 18942720-18944110 |
| 76 | *P. deltoides* | I | PdGATA5 | Podel.04G165400.1 | 327 | 2 | Chr04 | 17937941-17939017 |
| 77 | *P. deltoides* | I | PdGATA6a | Podel.04G217800.1 | 301 | 3 | Chr04 | 21943842-21947991 |
| 78 | *P. deltoides* | I | PdGATA6b | Podel.04G217800.2 | 301 | 3 | Chr04 | 21943842-21947991 |
| 79 | *P. deltoides* | I | PdGATA6c | Podel.04G217800.3 | 295 | 3 | Chr04 | 21943842-21947991 |
| 80 | *P. deltoides* | I | PdGATA6d | Podel.04G217800.4 | 295 | 3 | Chr04 | 21943842-21947991 |
| 81 | *P. deltoides* | I | PdGATA6e | Podel.04G217800.5 | 301 | 3 | Chr04 | 21943842-21947991 |
| 82 | *P. deltoides* | I | PdGATA6f | Podel.04G217800.6 | 232 | 1 | Chr04 | 21943842-21944540 |
| 83 | *P. deltoides* | I | PdGATA6g | Podel.04G217800.7 | 301 | 3 | Chr04 | 21943842-21947991 |
| 84 | *P. deltoides* | I | PdGATA6h | Podel.04G217800.8 | 301 | 3 | Chr04 | 21943842-21947218 |
| 85 | *P. deltoides* | I | PdGATA6i | Podel.04G217800.9 | 295 | 3 | Chr04 | 21943842-21947991 |
| 86 | *P. deltoides* | I | PdGATA7 | Podel.05G072100.1 | 233 | 2 | Chr05 | 4872712-4873422 |
| 87 | *P. deltoides* | I | PdGATA8 | Podel.06G250500.1 | 373 | 2 | Chr06 | 25170590-25171853 |
| 88 | *P. deltoides* | I | PdGATA9 | Podel.07G018800.1 | 369 | 3 | Chr07 | 1466657-1467993 |
| 89 | *P. deltoides* | I | PdGATA10 | Podel.08G047500.1 | 354 | 2 | Chr08 | 3065740-3067473 |
| 90 | *P. deltoides* | I | PdGATA11a | Podel.09G128700.1 | 329 | 2 | Chr09 | 10607495-10608576 |
| 91 | *P. deltoides* | I | PdGATA11b | Podel.09G128700.2 | 329 | 2 | Chr09 | 10607495-10608576 |
| 92 | *P. deltoides* | I | PdGATA12 | Podel.10G228500.1 | 352 | 2 | Chr10 | 19854784-19856449 |
| 93 | *P. deltoides* | I | PdGATA13 | Podel.13G063200.1 | 295 | 2 | Chr13 | 4630733-4632032 |
| 94 | *P. deltoides* | I | PdGATA14 | Podel.14G062600.1 | 231 | 3 | Chr14 | 4593300-4594157 |
| 95 | *P. deltoides* | I | PdGATA15 | Podel.18G044500.1 | 380 | 2 | Chr18 | 3945695-3946967 |
| 96 | *P. deltoides* | I | PdGATA16 | Podel.19G031200.1 | 294 | 2 | Chr19 | 3339208-3340463 |
| 97 | *P. deltoides* | I | PdGATA17 | Podel.T008700.1 | 212 | 4 | scaffold_1043 | 11063-11768 |
| 98 | *P. deltoides* | I | PdGATA18 | Podel.T118100.1 | 82 | 1 | scaffold_280 | 3715-3963 |
| 99 | *P. deltoides* | II | PdGATA19 | Podel.02G222300.1 | 147 | 3 | Chr02 | 18335408-18336069 |
| 100 | *P. deltoides* | II | PdGATA20 | Podel.03G232100.1 | 226 | 3 | Chr03 | 22106055-22108025 |
| 101 | *P. deltoides* | II | PdGATA21 | Podel.05G020400.1 | 147 | 3 | Chr05 | 1539000-1540351 |
| 102 | *P. deltoides* | II | PdGATA22 | Podel.05G137000.1 | 254 | 2 | Chr05 | 10341405-10342273 |
| 103 | *P. deltoides* | II | PdGATA23 | Podel.06G241900.1 | 303 | 3 | Chr06 | 24518631-24520071 |
| 104 | *P. deltoides* | II | PdGATA24 | Podel.07G029600.1 | 254 | 2 | Chr07 | 2326082-2326980 |
| 105 | *P. deltoides* | II | PdGATA25 | Podel.08G242600.1 | 134 | 3 | Chr08 | 19305764-19306366 |
| 106 | *P. deltoides* | II | PdGATA26 | Podel.10G001200.1 | 148 | 3 | Chr10 | 160700-161339 |
| 107 | *P. deltoides* | II | PdGATA27 | Podel.14G129000.1 | 133 | 3 | Chr14 | 9458187-9458791 |
| 108 | *P. deltoides* | III | PdGATA28a | Podel.02G121100.1 | 359 | 10 | Chr02 | 9082728-9087458 |
| 109 | *P. deltoides* | III | PdGATA28b | Podel.02G121100.2 | 285 | 8 | Chr02 | 9083794-9087458 |
| 110 | *P. deltoides* | III | PdGATA29 | Podel.02G121200.1 | 290 | 7 | Chr02 | 9090792-9095358 |
| 111 | *P. deltoides* | III | PdGATA30 | Podel.05G164200.1 | 371 | 10 | Chr05 | 14919219-14923811 |
| 112 | *P. deltoides* | III | PdGATA31 | Podel.05G164400.1 | 284 | 7 | Chr05 | 14981537-14993415 |
| 113 | *P. deltoides* | III | PdGATA32 | Podel.07G126800.1 | 318 | 7 | Chr07 | 14141687-14144287 |
| 114 | *P. deltoides* | III | PdGATA33a | Podel.07G126900.1 | 383 | 11 | Chr07 | 14147214-14152638 |
| 115 | *P. deltoides* | III | PdGATA33b | Podel.07G126900.2 | 361 | 10 | Chr07 | 14147214-14152638 |
| 116 | *P. deltoides* | III | PdGATA34a | Podel.10G259300.1 | 307 | 7 | Chr10 | 21617436-21620253 |
| 117 | *P. deltoides* | III | PdGATA34b | Podel.10G259300.2 | 307 | 7 | Chr10 | 21617436-21620253 |
| 118 | *P. deltoides* | III | PdGATA35a | Podel.17G043000.1 | 379 | 11 | Chr17 | 3685891-3690790 |
| 119 | *P. deltoides* | III | PdGATA35b | Podel.17G043000.2 | 368 | 10 | Chr17 | 3686036-3690790 |
| 120 | *P. deltoides* | III | PdGATA36 | Podel.17G043100.1 | 86 | 3 | Chr17 | 3702022-3704270 |
| 121 | *P. deltoides* | IV | PdGATA37 | Podel.01G163400.1 | 545 | 8 | Chr01 | 13533726-13539074 |
| 122 | *P. deltoides* | IV | PdGATA38 | Podel.03G086300.1 | 540 | 8 | Chr03 | 10636864-10642152 |
| 123 | *P. euphratica* | I | PeGATA1 | rna15028 | 370 | 3 | NW_011499868.1 | 1728861-1730196 |
| 124 | *P. euphratica* | I | PeGATA2 | rna1824 | 331 | 2 | NW_011499846.1 | 265816-266899 |
| 125 | *P. euphratica* | I | PeGATA3 | rna20562 | 232 | 3 | NW_011499886.1 | 1183451-1186648 |
| 126 | *P. euphratica* | I | PeGATA4 | rna21162 | 358 | 2 | NW_011499888.1 | 1177221-1178995 |
| 127 | *P. euphratica* | I | PeGATA5 | rna2321 | 247 | 3 | NW_011499846.1 | 3036868-3037762 |
| 128 | *P. euphratica* | I | PeGATA6 | rna23780 | 298 | 2 | NW_011499899.1 | 639749-641062 |
| 129 | *P. euphratica* | I | PeGATA7 | rna26731 | 328 | 2 | NW_011499915.1 | 256097-257174 |
| 130 | *P. euphratica* | I | PeGATA8a | rna30489 | 314 | 3 | NW_011499941.1 | 520253-523629 |
| 131 | *P. euphratica* | I | PeGATA8b | rna30490 | 320 | 3 | NW_011499941.1 | 520253-523629 |
| 132 | *P. euphratica* | I | PeGATA9a | rna30631 | 355 | 2 | NW_011499942.1 | 300434-302110 |
| 133 | *P. euphratica* | I | PeGATA9b | rna30632 | 355 | 2 | NW_011499942.1 | 300434-302110 |
| 134 | *P. euphratica* | I | PeGATA10 | rna36660 | 308 | 2 | NW_011500008.1 | 93763-94866 |
| 135 | *P. euphratica* | I | PeGATA11 | rna3782 | 329 | 2 | NW_011499847.1 | 2387658-2388740 |
| 136 | *P. euphratica* | I | PeGATA12 | rna41866 | 256 | 2 | NW_011500094.1 | 217181-218572 |
| 137 | *P. euphratica* | I | PeGATA13 | rna50055 | 294 | 2 | NW_011500500.1 | 61217-62430 |
| 138 | *P. euphratica* | I | PeGATA14 | rna52191 | 256 | 2 | NW_011500832.1 | 24185-25576 |
| 139 | *P. euphratica* | I | PeGATA15 | rna54965 | 380 | 2 | NW_011501903.1 | 14431-15702 |
| 140 | *P. euphratica* | I | PeGATA16 | rna6430 | 246 | 2 | NW_011499849.1 | 4117124-4118014 |
| 141 | *P. euphratica* | I | PeGATA17 | rna8595 | 373 | 2 | NW_011499853.1 | 2162936-2164197 |
| 142 | *P. euphratica* | I | PeGATA18 | rna9703 | 258 | 2 | NW_011499855.1 | 1722691-1724099 |
| 143 | *P. euphratica* | II | PeGATA19 | rna14322 | 383 | 4 | NW_011499866.1 | 708857-714745 |
| 144 | *P. euphratica* | II | PeGATA20 | rna14323 | 238 | 3 | NW_011499866.1 | 717953-719908 |
| 145 | *P. euphratica* | II | PeGATA21 | rna14926 | 254 | 2 | NW_011499868.1 | 1057534-1058436 |
| 146 | *P. euphratica* | II | PeGATA22 | rna26461 | 161 | 3 | NW_011499913.1 | 897898-899039 |
| 147 | *P. euphratica* | II | PeGATA23 | rna27493 | 147 | 2 | NW_011499919.1 | 728112-728689 |
| 148 | *P. euphratica* | II | PeGATA24a | rna31876 | 145 | 3 | NW_011499951.1 | 66016-66671 |
| 149 | *P. euphratica* | II | PeGATA24b | rna31877 | 145 | 3 | NW_011499951.1 | 66016-66671 |
| 150 | *P. euphratica* | II | PeGATA25 | rna3234 | 133 | 3 | NW_011499846.1 | 7842482-7843086 |
| 151 | *P. euphratica* | II | PeGATA26a | rna36298 | 148 | 3 | NW_011500004.1 | 103697-104336 |
| 152 | *P. euphratica* | II | PeGATA26b | rna36299 | 149 | 3 | NW_011500004.1 | 103697-104336 |
| 153 | *P. euphratica* | II | PeGATA27 | rna40162 | 139 | 3 | NW_011500061.1 | 40494-41118 |
| 154 | *P. euphratica* | II | PeGATA28 | rna43512 | 254 | 2 | NW_011500132.1 | 138800-139668 |
| 155 | *P. euphratica* | II | PeGATA29 | rna50676 | 302 | 3 | NW_011500584.1 | 13080-14502 |
| 156 | *P. euphratica* | II | PeGATA30 | rna8460 | 303 | 3 | NW_011499853.1 | 1450812-1452249 |
| 157 | *P. euphratica* | III | PeGATA31a | rna12133 | 305 | 6 | NW_011499860.1 | 2418821-2420835 |
| 158 | *P. euphratica* | III | PeGATA31b | rna12134 | 318 | 7 | NW_011499860.1 | 2418821-2421431 |
| 159 | *P. euphratica* | III | PeGATA32a | rna12135 | 362 | 10 | NW_011499860.1 | 2424696-2430248 |
| 160 | *P. euphratica* | III | PeGATA32b | rna12136 | 374 | 11 | NW_011499860.1 | 2424696-2430205 |
| 161 | *P. euphratica* | III | PeGATA32c | rna12137 | 384 | 11 | NW_011499860.1 | 2424696-2430248 |
| 162 | *P. euphratica* | III | PeGATA33a | rna32812 | 383 | 11 | NW_011499960.1 | 36051-40926 |
| 163 | *P. euphratica* | III | PeGATA33b | rna32813 | 386 | 11 | NW_011499960.1 | 36051-40926 |
| 164 | *P. euphratica* | III | PeGATA34 | rna36445 | 307 | 7 | NW_011500005.1 | 367168-369938 |
| 165 | *P. euphratica* | III | PeGATA35a | rna46041 | 381 | 10 | NW_011500219.1 | 224604-229229 |
| 166 | *P. euphratica* | III | PeGATA35b | rna46042 | 368 | 10 | NW_011500219.1 | 224604-228777 |
| 167 | *P. euphratica* | III | PeGATA35c | rna46043 | 381 | 10 | NW_011500219.1 | 224604-229229 |
| 168 | *P. euphratica* | III | PeGATA35d | rna46044 | 367 | 10 | NW_011500219.1 | 224604-229229 |
| 169 | *P. euphratica* | III | PeGATA35e | rna46045 | 365 | 10 | NW_011500219.1 | 224604-229229 |
| 170 | *P. euphratica* | III | PeGATA35f | rna46046 | 324 | 8 | NW_011500219.1 | 224604-228266 |
| 171 | *P. euphratica* | III | PeGATA36 | rna53960 | 288 | 7 | NW_011501398.1 | 20674-32221 |
| 172 | *P. euphratica* | III | PeGATA37a | rna5939 | 357 | 10 | NW_011499849.1 | 1561048-1565687 |
| 173 | *P. euphratica* | III | PeGATA37b | rna5940 | 357 | 10 | NW_011499849.1 | 1561048-1565687 |
| 174 | *P. euphratica* | III | PeGATA37c | rna5941 | 333 | 9 | NW_011499849.1 | 1561824-1565687 |
| 175 | *P. euphratica* | III | PeGATA38 | rna5942 | 290 | 7 | NW_011499849.1 | 1569105-1573860 |
| 176 | *P. euphratica* | IV | PeGATA39 | rna21402 | 540 | 8 | NW_011499889.1 | 978922-984137 |
| 177 | *P. euphratica* | IV | PeGATA40 | rna37522 | 545 | 8 | NW_011500021.1 | 160655-165986 |
| 178 | *P. pruinosa* | I | PpGATA1 | Ppr_1544.6400 | 250 | 2 | scaffold1544 | 3825704-3826558 |
| 179 | *P. pruinosa* | I | PpGATA2 | Ppr_1545.6670 | 298 | 2 | scaffold1545 | 1332252-1333562 |
| 180 | *P. pruinosa* | I | PpGATA3 | Ppr_1581.7150 | 294 | 2 | scaffold1581 | 238660-239864 |
| 181 | *P. pruinosa* | I | PpGATA4 | Ppr_1614.7750 | 355 | 2 | scaffold1614 | 2925055-2926731 |
| 182 | *P. pruinosa* | I | PpGATA5 | Ppr_175.9008 | 372 | 3 | scaffold175 | 2049718-2051061 |
| 183 | *P. pruinosa* | I | PpGATA6 | Ppr_2025.10908 | 406 | 5 | scaffold2025 | 1968019-1980343 |
| 184 | *P. pruinosa* | I | PpGATA7 | Ppr_2264.12420 | 373 | 2 | scaffold2264 | 1621253-1622513 |
| 185 | *P. pruinosa* | I | PpGATA8 | Ppr_2457.14440 | 258 | 2 | scaffold2457 | 1913205-1914615 |
| 186 | *P. pruinosa* | I | PpGATA9 | Ppr_2464.14681 | 308 | 2 | scaffold2464 | 187670-188773 |
| 187 | *P. pruinosa* | I | PpGATA10 | Ppr_255.15000 | 329 | 2 | scaffold255 | 312041-313123 |
| 188 | *P. pruinosa* | I | PpGATA11 | Ppr_3320.17504 | 328 | 2 | scaffold3320 | 1095057-1096136 |
| 189 | *P. pruinosa* | I | PpGATA12 | Ppr_3355.18016 | 420 | 6 | scaffold3355 | 282530-289810 |
| 190 | *P. pruinosa* | I | PpGATA13 | Ppr_422.21224 | 266 | 3 | scaffold422 | 9161966-9163783 |
| 191 | *P. pruinosa* | I | PpGATA14 | Ppr_529.23273 | 380 | 2 | scaffold529 | 22335-23606 |
| 192 | *P. pruinosa* | I | PpGATA15 | Ppr_572.24526 | 258 | 2 | scaffold572 | 2497460-2498847 |
| 193 | *P. pruinosa* | I | PpGATA16 | Ppr_674.28393 | 358 | 2 | scaffold674 | 2314397-2316140 |
| 194 | *P. pruinosa* | I | PpGATA17 | Ppr_771.31193 | 187 | 1 | scaffold771 | 2326422-2326985 |
| 195 | *P. pruinosa* | II | PpGATA18 | Ppr_135944.2530 | 311 | 3 | scaffold135944 | 71268-72690 |
| 196 | *P. pruinosa* | II | PpGATA19 | Ppr_175.8933 | 225 | 2 | scaffold175 | 1413259-1414074 |
| 197 | *P. pruinosa* | II | PpGATA20 | Ppr_2714.15907 | 234 | 2 | scaffold2714 | 6796-7588 |
| 198 | *P. pruinosa* | II | PpGATA21 | Ppr_3489.18626 | 609 | 10 | scaffold3489 | 710583-718163 |
| 199 | *P. pruinosa* | II | PpGATA22 | Ppr_3489.18627 | 235 | 3 | scaffold3489 | 721345-723340 |
| 200 | *P. pruinosa* | II | PpGATA23 | Ppr_5277.23151 | 486 | 7 | scaffold5277 | 210872-226006 |
| 201 | *P. pruinosa* | II | PpGATA24 | Ppr_55019.23892 | 161 | 3 | scaffold55019 | 3668246-3669383 |
| 202 | *P. pruinosa* | II | PpGATA25 | Ppr_5571.24030 | 791 | 8 | scaffold5571 | 38402-46400 |
| 203 | *P. pruinosa* | II | PpGATA26 | Ppr_6607.26846 | 149 | 3 | scaffold6607 | 106131-106770 |
| 204 | *P. pruinosa* | II | PpGATA27 | Ppr_7389.30462 | 138 | 3 | scaffold7389 | 53601-54225 |
| 205 | *P. pruinosa* | II | PpGATA28 | Ppr_900.33792 | 200 | 4 | scaffold900 | 214151-215938 |
| 206 | *P. pruinosa* | III | PpGATA29 | Ppr_1422.5430 | 420 | 12 | scaffold1422 | 8579-13695 |
| 207 | *P. pruinosa* | III | PpGATA30 | Ppr_1614.8013 | 273 | 8 | scaffold1614 | 4572445-4575141 |
| 208 | *P. pruinosa* | III | PpGATA31 | Ppr_3340.17909 | 384 | 11 | scaffold3340 | 983243-988718 |
| 209 | *P. pruinosa* | III | PpGATA32 | Ppr_3340.17910 | 318 | 7 | scaffold3340 | 991992-994541 |
| 210 | *P. pruinosa* | III | PpGATA33 | Ppr_422.20977 | 357 | 10 | scaffold422 | 6682275-6686893 |
| 211 | *P. pruinosa* | III | PpGATA34 | Ppr_451.22081 | 338 | 11 | scaffold451 | 51155-55211 |
| 212 | *P. pruinosa* | III | PpGATA35 | Ppr_543.23390 | 279 | 7 | scaffold543 | 4726-16456 |
| 213 | *P. pruinosa* | IV | PpGATA36 | Ppr_139105.4373 | 545 | 8 | scaffold139105 | 124049-129332 |
| 214 | *P. pruinosa* | IV | PpGATA37 | Ppr_706.29385 | 537 | 7 | scaffold706 | 844703-848605 |
| 215 | *P. tremuloides* | I | PtsGATA1 | Potrs000805g01297.1 | 329 | 2 | Potrs000805 | 8273-9354 |
| 216 | *P. tremuloides* | I | PtsGATA2 | Potrs001087g33972.1 | 187 | 2 | Potrs001087 | 10910-11476 |
| 217 | *P. tremuloides* | I | PtsGATA3a | Potrs001239g30352.1 | 259 | 3 | Potrs001239 | 44304-45976 |
| 218 | *P. tremuloides* | I | PtsGATA3b | Potrs001239g30352.2 | 258 | 2 | Potrs001239 | 44304-45714 |
| 219 | *P. tremuloides* | I | PtsGATA4 | Potrs001341g02322.1 | 328 | 2 | Potrs001341 | 20223-21578 |
| 220 | *P. tremuloides* | I | PtsGATA5 | Potrs003048g04721.1 | 352 | 2 | Potrs003048 | 8076-9737 |
| 221 | *P. tremuloides* | I | PtsGATA6 | Potrs005410g07642.1 | 327 | 2 | Potrs005410 | 7287-8364 |
| 222 | *P. tremuloides* | I | PtsGATA7 | Potrs005530g07804.1 | 380 | 2 | Potrs005530 | 12574-13845 |
| 223 | *P. tremuloides* | I | PtsGATA8 | Potrs005636g07975.1 | 194 | 4 | Potrs005636 | 7996-8917 |
| 224 | *P. tremuloides* | I | PtsGATA9 | Potrs005869g08275.1 | 384 | 3 | Potrs005869 | 14198-17818 |
| 225 | *P. tremuloides* | I | PtsGATA10 | Potrs007926g10989.1 | 258 | 2 | Potrs007926 | 26801-28185 |
| 226 | *P. tremuloides* | I | PtsGATA11 | Potrs011539g16655.1 | 246 | 2 | Potrs011539 | 15847-16686 |
| 227 | *P. tremuloides* | I | PtsGATA12 | Potrs016791g19374.1 | 244 | 2 | Potrs016791 | 3905-4798 |
| 228 | *P. tremuloides* | I | PtsGATA13 | Potrs018071g20307.1 | 297 | 2 | Potrs018071 | 15449-16754 |
| 229 | *P. tremuloides* | I | PtsGATA14 | Potrs019343g21649.1 | 333 | 2 | Potrs019343 | 10465-11552 |
| 230 | *P. tremuloides* | I | PtsGATA15 | Potrs019807g22431.1 | 375 | 2 | Potrs019807 | 8813-10079 |
| 231 | *P. tremuloides* | I | PtsGATA16 | Potrs034935g23972.1 | 370 | 3 | Potrs034935 | 5142-6476 |
| 232 | *P. tremuloides* | I | PtsGATA17a | Potrs038654g24811.1 | 354 | 2 | Potrs038654 | 4002-5762 |
| 233 | *P. tremuloides* | I | PtsGATA17b | Potrs038654g24811.2 | 354 | 2 | Potrs038654 | 4002-5762 |
| 234 | *P. tremuloides* | II | PtsGATA18 | Potrs003807g05701.1 | 133 | 3 | Potrs003807 | 7456-8059 |
| 235 | *P. tremuloides* | II | PtsGATA19 | Potrs004003g05890.1 | 254 | 2 | Potrs004003 | 7061-7929 |
| 236 | *P. tremuloides* | II | PtsGATA20 | Potrs004402g06457.1 | 161 | 3 | Potrs004402 | 30849-31973 |
| 237 | *P. tremuloides* | II | PtsGATA21 | Potrs008806g12935.1 | 254 | 2 | Potrs008806 | 13392-14290 |
| 238 | *P. tremuloides* | II | PtsGATA22a | Potrs011072g16224.1 | 149 | 3 | Potrs011072 | 4548-5187 |
| 239 | *P. tremuloides* | II | PtsGATA22b | Potrs011072g16224.2 | 135 | 2 | Potrs011072 | 4684-5187 |
| 240 | *P. tremuloides* | II | PtsGATA23 | Potrs013552g17726.1 | 311 | 3 | Potrs013552 | 11910-13336 |
| 241 | *P. tremuloides* | II | PtsGATA24 | Potrs014906g18397.1 | 147 | 3 | Potrs014906 | 2690-3354 |
| 242 | *P. tremuloides* | II | PtsGATA25 | Potrs033562g23791.1 | 137 | 3 | Potrs033562 | 4884-5494 |
| 243 | *P. tremuloides* | II | PtsGATA26 | Potrs042419g26636.1 | 343 | 4 | Potrs042419 | 5514-7146 |
| 244 | *P. tremuloides* | III | PtsGATA27a | Potrs007869g10965.1 | 307 | 7 | Potrs007869 | 29760-32535 |
| 245 | *P. tremuloides* | III | PtsGATA27b | Potrs007869g10965.2 | 269 | 5 | Potrs007869 | 29760-31596 |
| 246 | *P. tremuloides* | III | PtsGATA28 | Potrs008504g12395.1 | 407 | 10 | Potrs008504 | 41449-46323 |
| 247 | *P. tremuloides* | III | PtsGATA29 | Potrs008504g34007.1 | 46 | 1 | Potrs008504 | 33765-33905 |
| 248 | *P. tremuloides* | III | PtsGATA30a | Potrs009269g14633.1 | 290 | 7 | Potrs009269 | 24721-29358 |
| 249 | *P. tremuloides* | III | PtsGATA30b | Potrs009269g14633.2 | 169 | 5 | Potrs009269 | 25425-29358 |
| 250 | *P. tremuloides* | III | PtsGATA31 | Potrs009269g14634.1 | 359 | 10 | Potrs009269 | 33463-38189 |
| 251 | *P. tremuloides* | III | PtsGATA32 | Potrs010767g15789.1 | 252 | 9 | Potrs010767 | 16997-20424 |
| 252 | *P. tremuloides* | III | PtsGATA33 | Potrs010767g15790.1 | 287 | 6 | Potrs010767 | 9101-11410 |
| 253 | *P. tremuloides* | III | PtsGATA34 | Potrs012774g17307.1 | 146 | 6 | Potrs012774 | 2863-7537 |
| 254 | *P. tremuloides* | III | PtsGATA35a | Potrs039377g25085.1 | 363 | 10 | Potrs039377 | 4446-9016 |
| 255 | *P. tremuloides* | III | PtsGATA35b | Potrs039377g25085.2 | 275 | 7 | Potrs039377 | 5611-9016 |
| 256 | *P. tremuloides* | IV | PtsGATA36a | Potrs001801g03140.1 | 553 | 9 | Potrs001801 | 5764-11820 |
| 257 | *P. tremuloides* | IV | PtsGATA36b | Potrs001801g03140.2 | 545 | 8 | Potrs001801 | 5764-11067 |
| 258 | *P. tremuloides* | IV | PtsGATA37 | Potrs013102g17508.1 | 540 | 8 | Potrs013102 | 4562-9835 |
| 259 | *P. tremula* | I | PtaGATA1 | Potra000194g00836.1 | 187 | 1 | Potra000194 | 80312-80875 |
| 260 | *P. tremula* | I | PtaGATA2a | Potra000350g01269.1 | 307 | 2 | Potra000350 | 17548-18644 |
| 261 | *P. tremula* | I | PtaGATA2b | Potra000350g01269.2 | 176 | 2 | Potra000350 | 17548-18150 |
| 262 | *P. tremula* | I | PtaGATA3a | Potra000395g01841.1 | 246 | 2 | Potra000395 | 202023-202863 |
| 263 | *P. tremula* | I | PtaGATA3b | Potra000395g01841.2 | 246 | 2 | Potra000395 | 202023-202863 |
| 264 | *P. tremula* | I | PtaGATA3c | Potra000395g01841.3 | 246 | 2 | Potra000395 | 202023-202863 |
| 265 | *P. tremula* | I | PtaGATA3d | Potra000395g01841.4 | 246 | 2 | Potra000395 | 202023-202863 |
| 266 | *P. tremula* | I | PtaGATA4 | Potra000561g04070.1 | 373 | 2 | Potra000561 | 65618-66878 |
| 267 | *P. tremula* | I | PtaGATA5a | Potra001402g11910.1 | 327 | 2 | Potra001402 | 60403-61479 |
| 268 | *P. tremula* | I | PtaGATA5b | Potra001402g11910.2 | 327 | 2 | Potra001402 | 60403-61479 |
| 269 | *P. tremula* | I | PtaGATA6a | Potra001479g12376.1 | 329 | 2 | Potra001479 | 37286-38368 |
| 270 | *P. tremula* | I | PtaGATA6b | Potra001479g12376.2 | 329 | 2 | Potra001479 | 37286-38368 |
| 271 | *P. tremula* | I | PtaGATA6c | Potra001479g12376.3 | 329 | 2 | Potra001479 | 37286-38368 |
| 272 | *P. tremula* | I | PtaGATA7 | Potra001956g15450.1 | 294 | 2 | Potra001956 | 49968-51223 |
| 273 | *P. tremula* | I | PtaGATA8a | Potra002063g16095.1 | 354 | 2 | Potra002063 | 116113-118021 |
| 274 | *P. tremula* | I | PtaGATA8b | Potra002063g16095.2 | 354 | 2 | Potra002063 | 116113-118021 |
| 275 | *P. tremula* | I | PtaGATA8c | Potra002063g16095.3 | 354 | 2 | Potra002063 | 116113-118021 |
| 276 | *P. tremula* | I | PtaGATA8d | Potra002063g16095.4 | 354 | 2 | Potra002063 | 116113-118021 |
| 277 | *P. tremula* | I | PtaGATA9a | Potra002473g18725.1 | 259 | 3 | Potra002473 | 20248-21920 |
| 278 | *P. tremula* | I | PtaGATA9b | Potra002473g18725.2 | 258 | 2 | Potra002473 | 20510-21920 |
| 279 | *P. tremula* | I | PtaGATA10a | Potra003620g22193.1 | 386 | 2 | Potra003620 | 45707-46978 |
| 280 | *P. tremula* | I | PtaGATA10b | Potra003620g22193.2 | 380 | 2 | Potra003620 | 45707-46978 |
| 281 | *P. tremula* | I | PtaGATA11a | Potra003687g22443.1 | 258 | 2 | Potra003687 | 972-2355 |
| 282 | *P. tremula* | I | PtaGATA11b | Potra003687g22443.2 | 258 | 2 | Potra003687 | 972-2355 |
| 283 | *P. tremula* | I | PtaGATA12 | Potra003793g22879.1 | 246 | 2 | Potra003793 | 45817-46716 |
| 284 | *P. tremula* | I | PtaGATA13a | Potra003949g23719.1 | 352 | 2 | Potra003949 | 54991-56656 |
| 285 | *P. tremula* | I | PtaGATA13b | Potra003949g23719.2 | 352 | 2 | Potra003949 | 54991-56656 |
| 286 | *P. tremula* | I | PtaGATA13c | Potra003949g23719.3 | 352 | 2 | Potra003949 | 54991-56656 |
| 287 | *P. tremula* | I | PtaGATA14a | Potra004184g24670.1 | 370 | 3 | Potra004184 | 6356-7690 |
| 288 | *P. tremula* | I | PtaGATA14b | Potra004184g24670.2 | 338 | 2 | Potra004184 | 6585-7690 |
| 289 | *P. tremula* | I | PtaGATA15 | Potra004305g24793.1 | 293 | 2 | Potra004305 | 1066-2358 |
| 290 | *P. tremula* | I | PtaGATA16 | Potra009621g26398.1 | 316 | 2 | Potra009621 | 6064-9130 |
| 291 | *P. tremula* | I | PtaGATA17a | Potra188663g28622.1 | 330 | 2 | Potra188663 | 399-1476 |
| 292 | *P. tremula* | I | PtaGATA17b | Potra188663g28622.2 | 330 | 2 | Potra188663 | 399-1476 |
| 293 | *P. tremula* | II | PtaGATA18 | Potra000433g02320.1 | 137 | 3 | Potra000433 | 201684-202294 |
| 294 | *P. tremula* | II | PtaGATA19a | Potra001085g09392.1 | 154 | 2 | Potra001085 | 42381-42958 |
| 295 | *P. tremula* | II | PtaGATA19b | Potra001085g09392.2 | 147 | 3 | Potra001085 | 42301-42958 |
| 296 | *P. tremula* | II | PtaGATA20 | Potra001232g10597.1 | 161 | 3 | Potra001232 | 14697-15831 |
| 297 | *P. tremula* | II | PtaGATA21 | Potra001490g12434.1 | 148 | 3 | Potra001490 | 7131-7770 |
| 298 | *P. tremula* | II | PtaGATA22 | Potra002285g17501.1 | 256 | 2 | Potra002285 | 14644-15518 |
| 299 | *P. tremula* | II | PtaGATA23a | Potra002415g18371.1 | 312 | 3 | Potra002415 | 16657-18085 |
| 300 | *P. tremula* | II | PtaGATA23b | Potra002415g18371.2 | 303 | 3 | Potra002415 | 16657-18085 |
| 301 | *P. tremula* | II | PtaGATA24 | Potra168149g27342.1 | 133 | 3 | Potra168149 | 533-1135 |
| 302 | *P. tremula* | III | PtaGATA25a | Potra001342g11505.1 | 307 | 7 | Potra001342 | 110547-113308 |
| 303 | *P. tremula* | III | PtaGATA25b | Potra001342g11505.2 | 301 | 6 | Potra001342 | 110547-113186 |
| 304 | *P. tremula* | III | PtaGATA26a | Potra001373g11735.1 | 363 | 10 | Potra001373 | 76207-80755 |
| 305 | *P. tremula* | III | PtaGATA26b | Potra001373g11735.2 | 343 | 9 | Potra001373 | 76781-80755 |
| 306 | *P. tremula* | III | PtaGATA26c | Potra001373g11735.3 | 343 | 9 | Potra001373 | 76781-80755 |
| 307 | *P. tremula* | III | PtaGATA26d | Potra001373g11735.4 | 313 | 7 | Potra001373 | 77229-80755 |
| 308 | *P. tremula* | III | PtaGATA26e | Potra001373g11735.5 | 289 | 8 | Potra001373 | 77197-80755 |
| 309 | *P. tremula* | III | PtaGATA26f | Potra001373g11735.6 | 250 | 9 | Potra001373 | 76207-79880 |
| 310 | *P. tremula* | III | PtaGATA26g | Potra001373g11735.7 | 188 | 7 | Potra001373 | 76207-79116 |
| 311 | *P. tremula* | III | PtaGATA27 | Potra001952g15419.1 | 389 | 9 | Potra001952 | 32912-37418 |
| 312 | *P. tremula* | III | PtaGATA28 | Potra001952g15420.1 | 318 | 7 | Potra001952 | 40735-43311 |
| 313 | *P. tremula* | III | PtaGATA29 | Potra002524g19050.1 | 312 | 8 | Potra002524 | 54136-57853 |
| 314 | *P. tremula* | III | PtaGATA30a | Potra002524g19051.1 | 290 | 7 | Potra002524 | 61272-65845 |
| 315 | *P. tremula* | III | PtaGATA30b | Potra002524g19051.2 | 239 | 5 | Potra002524 | 63537-65845 |
| 316 | *P. tremula* | III | PtaGATA31 | Potra004074g24448.1 | 244 | 5 | Potra004074 | 3300-6340 |
| 317 | *P. tremula* | III | PtaGATA32 | Potra006684g25745.1 | 379 | 11 | Potra006684 | 2576-7466 |
| 318 | *P. tremula* | IV | PtaGATA33 | Potra001057g09047.1 | 550 | 8 | Potra001057 | 6990-12334 |
| 319 | *P. tremula* x *alba* | I | PtaaGATA1 | Potri.001G053500.1 | 336 | 2 | Chr01 | 4085585-4087215 |
| 320 | *P. tremula* x *alba* | I | PtaaGATA2a | Potri.001G188500.1 | 250 | 4 | Chr01 | 16653100-16654188 |
| 321 | *P. tremula* x *alba* | I | PtaaGATA2b | Potri.001G188500.2 | 200 | 1 | Chr01 | 16653100-16653702 |
| 322 | *P. tremula* x *alba* | I | PtaaGATA3 | Potri.002G142800.1 | 246 | 2 | Chr02 | 10594218-10595103 |
| 323 | *P. tremula* x *alba* | I | PtaaGATA4a | Potri.003G174800.1 | 259 | 3 | Chr03 | 18346747-18348416 |
| 324 | *P. tremula* x *alba* | I | PtaaGATA4b | Potri.003G174800.2 | 258 | 2 | Chr03 | 18346747-18348155 |
| 325 | *P. tremula* x *alba* | I | PtaaGATA5a | Potri.004G161500.1 | 327 | 2 | Chr04 | 18218619-18219692 |
| 326 | *P. tremula* x *alba* | I | PtaaGATA5b | Potri.004G161500.2 | 327 | 2 | Chr04 | 18218619-18219692 |
| 327 | *P. tremula* x *alba* | I | PtaaGATA6 | Potri.004G211800.1 | 302 | 3 | Chr04 | 21947497-21950889 |
| 328 | *P. tremula* x *alba* | I | PtaaGATA7 | Potri.005G066100.1 | 256 | 4 | Chr05 | 4765657-4768477 |
| 329 | *P. tremula* x *alba* | I | PtaaGATA8 | Potri.005G117600.1 | 333 | 2 | Chr05 | 9094249-9095346 |
| 330 | *P. tremula* x *alba* | I | PtaaGATA9a | Potri.006G237700.1 | 373 | 2 | Chr06 | 24690603-24691863 |
| 331 | *P. tremula* x *alba* | I | PtaaGATA9b | Potri.006G237700.2 | 357 | 3 | Chr06 | 24690603-24691863 |
| 332 | *P. tremula* x *alba* | I | PtaaGATA10 | Potri.007G016600.1 | 376 | 3 | Chr07 | 1263674-1265027 |
| 333 | *P. tremula* x *alba* | I | PtaaGATA11a | Potri.008G038900.1 | 354 | 2 | Chr08 | 2195007-2196749 |
| 334 | *P. tremula* x *alba* | I | PtaaGATA11b | Potri.008G038900.2 | 354 | 2 | Chr08 | 2195007-2196749 |
| 335 | *P. tremula* x *alba* | I | PtaaGATA11c | Potri.008G038900.3 | 354 | 2 | Chr08 | 2195007-2196749 |
| 336 | *P. tremula* x *alba* | I | PtaaGATA12a | Potri.009G123400.1 | 329 | 2 | Chr09 | 10280086-10281167 |
| 337 | *P. tremula* x *alba* | I | PtaaGATA12b | Potri.009G123400.2 | 329 | 2 | Chr09 | 10280086-10281167 |
| 338 | *P. tremula* x *alba* | I | PtaaGATA12c | Potri.009G123400.3 | 329 | 2 | Chr09 | 10280086-10281167 |
| 339 | *P. tremula* x *alba* | I | PtaaGATA13a | Potri.010G223300.2 | 352 | 2 | Chr10 | 20741238-20742896 |
| 340 | *P. tremula* x *alba* | I | PtaaGATA13b | Potri.010G223300.3 | 352 | 2 | Chr10 | 20741238-20742896 |
| 341 | *P. tremula* x *alba* | I | PtaaGATA14 | Potri.013G059600.1 | 295 | 2 | Chr13 | 4459252-4460553 |
| 342 | *P. tremula* x *alba* | I | PtaaGATA15 | Potri.014G058600.1 | 250 | 2 | Chr14 | 4539013-4539865 |
| 343 | *P. tremula* x *alba* | I | PtaaGATA16a | Potri.018G044900.1 | 380 | 2 | Chr18 | 4078025-4079296 |
| 344 | *P. tremula* x *alba* | I | PtaaGATA16b | Potri.018G044900.2 | 264 | 2 | Chr18 | 4078025-4079902 |
| 345 | *P. tremula* x *alba* | I | PtaaGATA16c | Potri.018G044900.3 | 353 | 2 | Chr18 | 4078025-4079296 |
| 346 | *P. tremula* x *alba* | I | PtaaGATA17 | Potri.019G033000.1 | 294 | 2 | Chr19 | 3788594-3789826 |
| 347 | *P. tremula* x *alba* | I | PtaaGATA18 | Potri.T158300.1 | 354 | 3 | scaffold_694 | 4641-5913 |
| 348 | *P. tremula* x *alba* | II | PtaaGATA19 | Potri.002G199800.1 | 147 | 3 | Chr02 | 16055736-16056397 |
| 349 | *P. tremula* x *alba* | II | PtaaGATA20 | Potri.003G213300.1 | 227 | 3 | Chr03 | 21112015-21113970 |
| 350 | *P. tremula* x *alba* | II | PtaaGATA21 | Potri.005G020500.1 | 161 | 3 | Chr05 | 1593531-1594687 |
| 351 | *P. tremula* x *alba* | II | PtaaGATA22 | Potri.005G122700.1 | 254 | 2 | Chr05 | 9547239-9548107 |
| 352 | *P. tremula* x *alba* | II | PtaaGATA23 | Potri.006G229200.1 | 355 | 4 | Chr06 | 24005480-24007971 |
| 353 | *P. tremula* x *alba* | II | PtaaGATA24 | Potri.007G024500.1 | 254 | 2 | Chr07 | 1849241-1850139 |
| 354 | *P. tremula* x *alba* | II | PtaaGATA25 | Potri.008G213900.1 | 139 | 3 | Chr08 | 16931331-16931949 |
| 355 | *P. tremula* x *alba* | II | PtaaGATA26a | Potri.010G001300.1 | 153 | 2 | Chr10 | 150155-150712 |
| 356 | *P. tremula* x *alba* | II | PtaaGATA26b | Potri.010G001300.2 | 149 | 3 | Chr10 | 150155-150794 |
| 357 | *P. tremula* x *alba* | II | PtaaGATA26c | Potri.010G001300.3 | 148 | 3 | Chr10 | 150155-150794 |
| 358 | *P. tremula* x *alba* | II | PtaaGATA26d | Potri.010G001300.4 | 139 | 4 | Chr10 | 150155-150794 |
| 359 | *P. tremula* x *alba* | II | PtaaGATA27 | Potri.014G124400.1 | 133 | 3 | Chr14 | 9592160-9592763 |
| 360 | *P. tremula* x *alba* | II | PtaaGATA28 | Potri.018G053600.1 | 303 | 3 | Chr18 | 5585949-5587382 |
| 361 | *P. tremula* x *alba* | III | PtaaGATA29a | Potri.002G110800.1 | 360 | 10 | Chr02 | 8195875-8200603 |
| 362 | *P. tremula* x *alba* | III | PtaaGATA29b | Potri.002G110800.2 | 272 | 9 | Chr02 | 8195875-8199673 |
| 363 | *P. tremula* x *alba* | III | PtaaGATA29c | Potri.002G110800.3 | 251 | 5 | Chr02 | 8198503-8200603 |
| 364 | *P. tremula* x *alba* | III | PtaaGATA30a | Potri.002G110900.1 | 290 | 7 | Chr02 | 8204006-8208686 |
| 365 | *P. tremula* x *alba* | III | PtaaGATA30b | Potri.002G110900.2 | 254 | 6 | Chr02 | 8204256-8208686 |
| 366 | *P. tremula* x *alba* | III | PtaaGATA30c | Potri.002G110900.3 | 261 | 6 | Chr02 | 8204231-8208686 |
| 367 | *P. tremula* x *alba* | III | PtaaGATA30d | Potri.002G110900.4 | 239 | 5 | Chr02 | 8206255-8208686 |
| 368 | *P. tremula* x *alba* | III | PtaaGATA31a | Potri.005G152500.1 | 365 | 10 | Chr05 | 14345346-14349916 |
| 369 | *P. tremula* x *alba* | III | PtaaGATA31b | Potri.005G152500.2 | 352 | 10 | Chr05 | 14345792-14349916 |
| 370 | *P. tremula* x *alba* | III | PtaaGATA31c | Potri.005G152500.3 | 352 | 10 | Chr05 | 14345792-14349916 |
| 371 | *P. tremula* x *alba* | III | PtaaGATA31d | Potri.005G152500.4 | 309 | 5 | Chr05 | 14347795-14349916 |
| 372 | *P. tremula* x *alba* | III | PtaaGATA32 | Potri.005G152800.1 | 288 | 7 | Chr05 | 14383561-14395349 |
| 373 | *P. tremula* x *alba* | III | PtaaGATA33 | Potri.007G116600.1 | 142 | 4 | Chr07 | 13728745-13730023 |
| 374 | *P. tremula* x *alba* | III | PtaaGATA34a | Potri.007G116700.1 | 383 | 11 | Chr07 | 13733091-13738476 |
| 375 | *P. tremula* x *alba* | III | PtaaGATA34b | Potri.007G116700.2 | 361 | 10 | Chr07 | 13733091-13738476 |
| 376 | *P. tremula* x *alba* | III | PtaaGATA34c | Potri.007G116700.3 | 372 | 10 | Chr07 | 13733091-13738326 |
| 377 | *P. tremula* x *alba* | III | PtaaGATA34d | Potri.007G116700.4 | 277 | 6 | Chr07 | 13733091-13736283 |
| 378 | *P. tremula* x *alba* | III | PtaaGATA35 | Potri.010G251600.1 | 307 | 7 | Chr10 | 22334861-22337680 |
| 379 | *P. tremula* x *alba* | III | PtaaGATA36a | Potri.017G042200.1 | 407 | 10 | Chr17 | 3570147-3575997 |
| 380 | *P. tremula* x *alba* | III | PtaaGATA36b | Potri.017G042200.2 | 383 | 11 | Chr17 | 3570002-3575895 |
| 381 | *P. tremula* x *alba* | III | PtaaGATA36c | Potri.017G042200.3 | 361 | 10 | Chr17 | 3570002-3575895 |
| 382 | *P. tremula* x *alba* | III | PtaaGATA36d | Potri.017G042200.4 | 397 | 10 | Chr17 | 3570147-3575997 |
| 383 | *P. tremula* x *alba* | III | PtaaGATA36e | Potri.017G042200.5 | 300 | 5 | Chr17 | 3572044-3575997 |
| 384 | *P. tremula* x *alba* | IV | PtaaGATA37a | Potri.001G151700.1 | 551 | 9 | Chr01 | 12499107-12504748 |
| 385 | *P. tremula* x *alba* | IV | PtaaGATA37b | Potri.001G151700.2 | 543 | 8 | Chr01 | 12499107-12504448 |
| 386 | *P. tremula* x *alba* | IV | PtaaGATA37c | Potri.001G151700.3 | 439 | 6 | Chr01 | 12500084-12504448 |
| 387 | *P. tremula* x *alba* | IV | PtaaGATA37d | Potri.001G151700.4 | 551 | 9 | Chr01 | 12499107-12504748 |
| 388 | *P. tremula* x *alba* | IV | PtaaGATA38a | Potri.003G082800.1 | 540 | 8 | Chr03 | 11052729-11058185 |
| 389 | *P. tremula* x *alba* | IV | PtaaGATA38b | Potri.003G082800.2 | 536 | 8 | Chr03 | 11052729-11058185 |
