# Supplementary material for: Genome-wide comparative analyses of GATA transcription factors among seven Populus genomes
Source: Sci Rep. 2021 Aug 16;11:16578. doi: 10.1038/s41598-021-95940-5 (PMC8367991; doi:10.1038/s41598-021-95940-5)
Supplement: Supplementary file 9 — Supplementary Information 9. [file 41598_2021_95940_MOESM9_ESM.docx]

**Table S4.** RNA-Seq data of *Populus* genus deposited in NCBI

| ***Populus* species name** | **# of experiments in SRA** | **# of runs in SRA** | **Total** **base pairs (bp)** |
| --- | --- | --- | --- |
| *Populus tremula* x *alba* | 26 | 152 | 674,199,571,770 |
| *Populus trichocarpa* | 1,631 | 1,721 | 7,453,709,975,626 |
| *Populus tremula* | 424 | 467 | 2,020,062,468,185 |
| *Populus deltoides* | 203 | 203 | 1,120,366,411,926 |
| *Populus tremuloides* | 43 | 79 | 620,313,793,670 |
| *Populus euphratica* | 33 | 86 | 1,046,992,949,635 |
| *Populus pruinosa* | 10 | 28 | 189,804,545,118 |
| **Total** | **2,370** | **2,736** | **13,125,449,715,930** |
