# Supplementary material for: Genome-wide comparative analyses of GATA transcription factors among seven Populus genomes
Source: Sci Rep. 2021 Aug 16;11:16578. doi: 10.1038/s41598-021-95940-5 (PMC8367991; doi:10.1038/s41598-021-95940-5)
Supplement: Supplementary file 10 — Supplementary Information 10. [file 41598_2021_95940_MOESM10_ESM.docx]

**Table S5.** Number of *Populus* GATA TFs without the GATA domain

| ***Populus* species name** | **GATA gene name** | **Number of GATA TFs without the GATA domain** | **Subfamily** | |  |
| --- | --- | --- | --- | --- | --- |
| *Populus tremula* x *alba* | PtaaGATA13 | 1 | I | |  |
|  | PtaaGATA21 | 1 | II | |  |
|  | PtaaGATA23 | 1 | II | |  |
|  | PtaaGATA27 | 1 | II | |  |
|  | PtaaGATA29 | 1 | III | |  |
|  | PtaaGATA31 | 1 | III | |  |
| *Populus tremula* | PtaGATA28 | 5 | III | |  |
|  | PtaGATA30 | 1 | III | |  |
|  | PtaGATA33 | 2 | IV | |  |
| *Populus tremuloides* | PtsGATA31 | 1 | III | |  |
|  | PtsGATA33 | 1 | III | |  |
|  | PtsGATA35 | 1 | III | |  |
| **Total** |  | **17** |  |  | |
