# Supplementary material for: Genome-wide comparative analyses of GATA transcription factors among seven Populus genomes
Source: Sci Rep. 2021 Aug 16;11:16578. doi: 10.1038/s41598-021-95940-5 (PMC8367991; doi:10.1038/s41598-021-95940-5)
Supplement: Supplementary file 11 — Supplementary Information 11. [file 41598_2021_95940_MOESM11_ESM.docx]

**Table S6.** Number of *Populus* GATA genes in each subfamily

| ***Populus* species name** | **Subfamily I** | | **Subfamily II** | | **Subfamily III** | | **Subfamily IV** | |
| --- | --- | --- | --- | --- | --- | --- | --- | --- |
|  | #of GATA genes | # of GATA TFs | #of GATA genes | # of GATA TFs | # of GATA genes | # of GATA TFs | # of GATA genes | # of GATA TFs |
| *Populus trichocarpa* | 18 | 35 | 10 | 12 | 9 | 15 | 2 | 5 |
| *Populus pruinosa* | 17 | 17 | 11 | 11 | 7 | 7 | 2 | 2 |
| *Populus euphratica* | 18 | 20 | 12 | 14 | 8 | 19 | 2 | 2 |
| *Populus deltoides* | 18 | 31 | 9 | 9 | 9 | 13 | 2 | 2 |
| *Populus tremuloides* | 17 | 19 | 9 | 10 | 9 | 12 | 2 | 3 |
| *Populus tremula* | 17 | 34 | 7 | 9 | 8 | 16 | 1 | 1 |
| *Populus tremula* x *alba* | 18 | 29 | 10 | 13 | 8 | 23 | 2 | 6 |
| **Total** | **123** | **185** | **68** | **78** | **58** | **105** | **13** | **21** |
