# Supplementary material for: Genome-wide comparative analyses of GATA transcription factors among seven Populus genomes
Source: Sci Rep. 2021 Aug 16;11:16578. doi: 10.1038/s41598-021-95940-5 (PMC8367991; doi:10.1038/s41598-021-95940-5)
Supplement: Supplementary file 12 — Supplementary Information 12. [file 41598_2021_95940_MOESM12_ESM.docx]

**Table S7.** List of identified GATA TFs of 21 *Populus* GATA gene clusters (PCs)

| **No** | **PC** | **Species** | **Sub-family** | **GATA name** | **Length (aa)** | **Domain type** |
| --- | --- | --- | --- | --- | --- | --- |
| 1 | 01 | *P. deltoides* | III | PdGATA28a | 359 | IVc |
| 2 | 01 | *P. deltoides* | III | PdGATA28b | 285 | IVc |
| 3 | 01 | *P. deltoides* | III | PdGATA30 | 371 | IVc |
| 4 | 01 | *P. deltoides* | III | PdGATA33a | 383 | IVc |
| 5 | 01 | *P. deltoides* | III | PdGATA33b | 361 | IVc |
| 6 | 01 | *P. deltoides* | III | PdGATA35a | 379 | IVc |
| 7 | 01 | *P. deltoides* | III | PdGATA35b | 368 | IVc |
| 8 | 01 | *P. euphratica* | III | PeGATA32a | 362 | IVc |
| 9 | 01 | *P. euphratica* | III | PeGATA32b | 374 | IVc |
| 10 | 01 | *P. euphratica* | III | PeGATA32c | 384 | IVc |
| 11 | 01 | *P. euphratica* | III | PeGATA33a | 383 | IVc |
| 12 | 01 | *P. euphratica* | III | PeGATA33b | 386 | IVc |
| 13 | 01 | *P. euphratica* | III | PeGATA35a | 381 | IVc |
| 14 | 01 | *P. euphratica* | III | PeGATA35b | 368 | IVc |
| 15 | 01 | *P. euphratica* | III | PeGATA35c | 381 | IVc |
| 16 | 01 | *P. euphratica* | III | PeGATA35d | 367 | IVc |
| 17 | 01 | *P. euphratica* | III | PeGATA35e | 365 | IVc |
| 18 | 01 | *P. euphratica* | III | PeGATA35f | 324 | IVc |
| 19 | 01 | *P. euphratica* | III | PeGATA37a | 357 | IVc |
| 20 | 01 | *P. euphratica* | III | PeGATA37b | 357 | IVc |
| 21 | 01 | *P. euphratica* | III | PeGATA37c | 333 | IVc |
| 22 | 01 | *P. pruinosa* | III | PpGATA29 | 420 | IVc |
| 23 | 01 | *P. pruinosa* | III | PpGATA31 | 384 | IVc |
| 24 | 01 | *P. pruinosa* | III | PpGATA33 | 357 | IVc |
| 25 | 01 | *P. pruinosa* | III | PpGATA34 | 338 | IVc |
| 26 | 01 | *P. tremula* x *alba* | III | PtaaGATA29a | 360 | IVc |
| 27 | 01 | *P. tremula* x *alba* | III | PtaaGATA29b | 272 | IVc |
| 28 | 01 | *P. tremula* x *alba* | III | PtaaGATA29c | 251 | IVc |
| 29 | 01 | *P. tremula* x *alba* | III | PtaaGATA31a | 365 | IVc |
| 30 | 01 | *P. tremula* x *alba* | III | PtaaGATA31b | 352 | IVc |
| 31 | 01 | *P. tremula* x *alba* | III | PtaaGATA31c | 352 | IVc |
| 32 | 01 | *P. tremula* x *alba* | III | PtaaGATA31d | 309 | IVc |
| 33 | 01 | *P. tremula* x *alba* | III | PtaaGATA34a | 383 | IVc |
| 34 | 01 | *P. tremula* x *alba* | III | PtaaGATA34b | 361 | IVc |
| 35 | 01 | *P. tremula* x *alba* | III | PtaaGATA34c | 372 | IVc |
| 36 | 01 | *P. tremula* x *alba* | III | PtaaGATA34d | 277 | IVc |
| 37 | 01 | *P. tremula* x *alba* | III | PtaaGATA36a | 407 | IVc |
| 38 | 01 | *P. tremula* x *alba* | III | PtaaGATA36b | 383 | IVc |
| 39 | 01 | *P. tremula* x *alba* | III | PtaaGATA36c | 361 | IVc |
| 40 | 01 | *P. tremula* x *alba* | III | PtaaGATA36d | 397 | IVp |
| 41 | 01 | *P. tremula* x *alba* | III | PtaaGATA36e | 300 | IVc |
| 42 | 01 | *P. tremula* | III | PtaGATA26a | 363 | IVc |
| 43 | 01 | *P. tremula* | III | PtaGATA26b | 343 | IVc |
| 44 | 01 | *P. tremula* | III | PtaGATA26c | 343 | IVc |
| 45 | 01 | *P. tremula* | III | PtaGATA26d | 313 | IVc |
| 46 | 01 | *P. tremula* | III | PtaGATA26e | 289 | IVc |
| 47 | 01 | *P. tremula* | III | PtaGATA26f | 250 | IVc |
| 48 | 01 | *P. tremula* | III | PtaGATA26g | 188 | IVc |
| 49 | 01 | *P. tremula* | III | PtaGATA27 | 389 | IVc |
| 50 | 01 | *P. tremula* | III | PtaGATA29 | 312 | IVc |
| 51 | 01 | *P. tremula* | III | PtaGATA32 | 379 | IVc |
| 52 | 01 | *P. trichocarpa* | III | PtrGATA17a | 365 | IVc |
| 53 | 01 | *P. trichocarpa* | III | PtrGATA17b | 324 | IVc |
| 54 | 01 | *P. trichocarpa* | III | PtrGATA17c | 321 | IVc |
| 55 | 01 | *P. trichocarpa* | III | PtrGATA24a | 384 | IVc |
| 56 | 01 | *P. trichocarpa* | III | PtrGATA24b | 362 | IVc |
| 57 | 01 | *P. trichocarpa* | III | PtrGATA34a | 383 | IVc |
| 58 | 01 | *P. trichocarpa* | III | PtrGATA34b | 361 | IVc |
| 59 | 01 | *P. trichocarpa* | III | PtrGATA34c | 372 | IVc |
| 60 | 01 | *P. trichocarpa* | III | PtrGATA4a | 360 | IVc |
| 61 | 01 | *P. trichocarpa* | III | PtrGATA4b | 338 | IVc |
| 62 | 01 | *P. tremuloides* | III | PtsGATA28 | 407 | IVc |
| 63 | 01 | *P. tremuloides* | III | PtsGATA31 | 359 | IVc |
| 64 | 01 | *P. tremuloides* | III | PtsGATA32 | 252 | IVc |
| 65 | 01 | *P. tremuloides* | III | PtsGATA35a | 363 | IVc |
| 66 | 01 | *P. tremuloides* | III | PtsGATA35b | 275 | IVc |
| 67 | 02 | *P. deltoides* | III | PdGATA32 | 318 | IVc |
| 68 | 02 | *P. deltoides* | III | PdGATA36 | 86 | IVp |
| 69 | 02 | *P. euphratica* | III | PeGATA31a | 305 | IVc |
| 70 | 02 | *P. euphratica* | III | PeGATA31b | 318 | IVc |
| 71 | 02 | *P. pruinosa* | III | PpGATA32 | 318 | IVc |
| 72 | 02 | *P. tremula* x *alba* | III | PtaaGATA33 | 142 | IVc |
| 73 | 02 | *P. tremula* | III | PtaGATA28 | 318 | IVc |
| 74 | 02 | *P. trichocarpa* | III | PtrGATA23 | 142 | IVc |
| 75 | 02 | *P. trichocarpa* | III | PtrGATA35 | 100 | IVc |
| 76 | 02 | *P. tremuloides* | III | PtsGATA29 | 46 | IVp |
| 77 | 02 | *P. tremuloides* | III | PtsGATA33 | 287 | IVc |
| 78 | 03 | *P. deltoides* | III | PdGATA29 | 290 | IVc |
| 79 | 03 | *P. deltoides* | III | PdGATA31 | 284 | IVc |
| 80 | 03 | *P. euphratica* | III | PeGATA36 | 288 | IVc |
| 81 | 03 | *P. euphratica* | III | PeGATA38 | 290 | IVc |
| 82 | 03 | *P. pruinosa* | III | PpGATA35 | 279 | IVc |
| 83 | 03 | *P. tremula* x *alba* | III | PtaaGATA30a | 290 | IVc |
| 84 | 03 | *P. tremula* x *alba* | III | PtaaGATA30b | 254 | IVc |
| 85 | 03 | *P. tremula* x *alba* | III | PtaaGATA30c | 261 | IVc |
| 86 | 03 | *P. tremula* x *alba* | III | PtaaGATA30d | 239 | IVc |
| 87 | 03 | *P. tremula* x *alba* | III | PtaaGATA32 | 288 | IVc |
| 88 | 03 | *P. tremula* | III | PtaGATA30a | 290 | IVc |
| 89 | 03 | *P. tremula* | III | PtaGATA30b | 239 | IVc |
| 90 | 03 | *P. tremula* | III | PtaGATA31 | 244 | IVc |
| 91 | 03 | *P. trichocarpa* | III | PtrGATA18 | 288 | IVc |
| 92 | 03 | *P. trichocarpa* | III | PtrGATA5 | 290 | IVc |
| 93 | 03 | *P. tremuloides* | III | PtsGATA30a | 290 | IVc |
| 94 | 03 | *P. tremuloides* | III | PtsGATA30b | 169 | IVc |
| 95 | 03 | *P. tremuloides* | III | PtsGATA34 | 146 | IVc |
| 96 | 04 | *P. deltoides* | III | PdGATA34a | 307 | IVc |
| 97 | 04 | *P. deltoides* | III | PdGATA34b | 307 | IVc |
| 98 | 04 | *P. euphratica* | III | PeGATA34 | 307 | IVc |
| 99 | 04 | *P. pruinosa* | III | PpGATA30 | 273 | IVc |
| 100 | 04 | *P. tremula* x *alba* | III | PtaaGATA35 | 307 | IVc |
| 101 | 04 | *P. tremula* | III | PtaGATA25a | 307 | IVc |
| 102 | 04 | *P. tremula* | III | PtaGATA25b | 301 | IVc |
| 103 | 04 | *P. trichocarpa* | III | PtrGATA30 | 307 | IVc |
| 104 | 04 | *P. tremuloides* | III | PtsGATA27a | 307 | IVc |
| 105 | 04 | *P. tremuloides* | III | PtsGATA27b | 269 | IVc |
| 106 | 05 | *P. deltoides* | IV | PdGATA37 | 545 | IVb |
| 107 | 05 | *P. deltoides* | IV | PdGATA38 | 540 | IVb |
| 108 | 05 | *P. euphratica* | IV | PeGATA39 | 540 | IVb |
| 109 | 05 | *P. euphratica* | IV | PeGATA40 | 545 | IVb |
| 110 | 05 | *P. pruinosa* | IV | PpGATA36 | 545 | IVb |
| 111 | 05 | *P. pruinosa* | IV | PpGATA37 | 537 | IVp |
| 112 | 05 | *P. tremula* x *alba* | IV | PtaaGATA37a | 551 | IVb |
| 113 | 05 | *P. tremula* x *alba* | IV | PtaaGATA37b | 543 | IVb |
| 114 | 05 | *P. tremula* x *alba* | IV | PtaaGATA37c | 439 | IVb |
| 115 | 05 | *P. tremula* x *alba* | IV | PtaaGATA37d | 551 | IVb |
| 116 | 05 | *P. tremula* x *alba* | IV | PtaaGATA38a | 540 | IVb |
| 117 | 05 | *P. tremula* x *alba* | IV | PtaaGATA38b | 536 | IVb |
| 118 | 05 | *P. tremula* | IV | PtaGATA33 | 550 | IVb |
| 119 | 05 | *P. trichocarpa* | IV | PtrGATA2a | 544 | IVb |
| 120 | 05 | *P. trichocarpa* | IV | PtrGATA2b | 552 | IVb |
| 121 | 05 | *P. trichocarpa* | IV | PtrGATA2c | 550 | IVb |
| 122 | 05 | *P. trichocarpa* | IV | PtrGATA8a | 540 | IVb |
| 123 | 05 | *P. trichocarpa* | IV | PtrGATA8b | 527 | IVb |
| 124 | 05 | *P. tremuloides* | IV | PtsGATA36a | 553 | IVb |
| 125 | 05 | *P. tremuloides* | IV | PtsGATA36b | 545 | IVb |
| 126 | 05 | *P. tremuloides* | IV | PtsGATA37 | 540 | IVb |
| 127 | 06 | *P. deltoides* | II | PdGATA19 | 147 | IVb |
| 128 | 06 | *P. deltoides* | II | PdGATA27 | 133 | IVb |
| 129 | 06 | *P. euphratica* | II | PeGATA24a | 145 | IVb |
| 130 | 06 | *P. euphratica* | II | PeGATA24b | 145 | IVb |
| 131 | 06 | *P. euphratica* | II | PeGATA25 | 133 | IVb |
| 132 | 06 | *P. pruinosa* | II | PpGATA25 | 791 | IVb |
| 133 | 06 | *P. pruinosa* | II | PpGATA28 | 200 | IVb |
| 134 | 06 | *P. tremula* x *alba* | II | PtaaGATA19 | 147 | IVb |
| 135 | 06 | *P. tremula* x *alba* | II | PtaaGATA27 | 133 | IVb |
| 136 | 06 | *P. tremula* | II | PtaGATA19a | 154 | IVb |
| 137 | 06 | *P. tremula* | II | PtaGATA19b | 147 | IVb |
| 138 | 06 | *P. tremula* | II | PtaGATA24 | 133 | IVb |
| 139 | 06 | *P. trichocarpa* | II | PtrGATA33 | 133 | IVb |
| 140 | 06 | *P. trichocarpa* | II | PtrGATA7 | 147 | IVb |
| 141 | 06 | *P. tremuloides* | II | PtsGATA18 | 133 | IVb |
| 142 | 06 | *P. tremuloides* | II | PtsGATA24 | 147 | IVb |
| 143 | 07 | *P. deltoides* | II | PdGATA21 | 147 | IVp |
| 144 | 07 | *P. euphratica* | II | PeGATA22 | 161 | IVb |
| 145 | 07 | *P. pruinosa* | II | PpGATA24 | 161 | IVb |
| 146 | 07 | *P. tremula* x *alba* | II | PtaaGATA21 | 161 | IVb |
| 147 | 07 | *P. tremula* | II | PtaGATA20 | 161 | IVb |
| 148 | 07 | *P. trichocarpa* | II | PtrGATA13 | 161 | IVb |
| 149 | 07 | *P. tremuloides* | II | PtsGATA20 | 161 | IVb |
| 150 | 08 | *P. deltoides* | II | PdGATA25 | 134 | IVb |
| 151 | 08 | *P. deltoides* | II | PdGATA26 | 148 | IVb |
| 152 | 08 | *P. euphratica* | II | PeGATA26a | 148 | IVb |
| 153 | 08 | *P. euphratica* | II | PeGATA26b | 149 | IVb |
| 154 | 08 | *P. euphratica* | II | PeGATA27 | 139 | IVb |
| 155 | 08 | *P. pruinosa* | II | PpGATA26 | 149 | IVb |
| 156 | 08 | *P. pruinosa* | II | PpGATA27 | 138 | IVb |
| 157 | 08 | *P. tremula* x *alba* | II | PtaaGATA25 | 139 | IVb |
| 158 | 08 | *P. tremula* x *alba* | II | PtaaGATA26a | 153 | IVb |
| 159 | 08 | *P. tremula* x *alba* | II | PtaaGATA26b | 149 | IVb |
| 160 | 08 | *P. tremula* x *alba* | II | PtaaGATA26c | 148 | IVb |
| 161 | 08 | *P. tremula* x *alba* | II | PtaaGATA26d | 139 | IVb |
| 162 | 08 | *P. tremula* | II | PtaGATA18 | 137 | IVb |
| 163 | 08 | *P. tremula* | II | PtaGATA21 | 148 | IVb |
| 164 | 08 | *P. trichocarpa* | II | PtrGATA26 | 138 | IVb |
| 165 | 08 | *P. trichocarpa* | II | PtrGATA28a | 153 | IVb |
| 166 | 08 | *P. trichocarpa* | II | PtrGATA28b | 149 | IVb |
| 167 | 08 | *P. trichocarpa* | II | PtrGATA28c | 148 | IVb |
| 168 | 08 | *P. tremuloides* | II | PtsGATA22a | 149 | IVb |
| 169 | 08 | *P. tremuloides* | II | PtsGATA22b | 135 | IVb |
| 170 | 08 | *P. tremuloides* | II | PtsGATA25 | 137 | IVb |
| 171 | 09 | *P. deltoides* | II | PdGATA23 | 303 | IVb |
| 172 | 09 | *P. euphratica* | II | PeGATA29 | 302 | IVb |
| 173 | 09 | *P. euphratica* | II | PeGATA30 | 303 | IVb |
| 174 | 09 | *P. pruinosa* | II | PpGATA18 | 311 | IVb |
| 175 | 09 | *P. pruinosa* | II | PpGATA20 | 234 | IVb |
| 176 | 09 | *P. tremula* x *alba* | II | PtaaGATA23 | 355 | IVb |
| 177 | 09 | *P. tremula* x *alba* | II | PtaaGATA28 | 303 | IVb |
| 178 | 09 | *P. tremula* | II | PtaGATA23a | 312 | IVb |
| 179 | 09 | *P. tremula* | II | PtaGATA23b | 303 | IVb |
| 180 | 09 | *P. trichocarpa* | II | PtrGATA19 | 303 | IVb |
| 181 | 09 | *P. trichocarpa* | II | PtrGATA37 | 303 | IVb |
| 182 | 09 | *P. tremuloides* | II | PtsGATA23 | 311 | IVb |
| 183 | 09 | *P. tremuloides* | II | PtsGATA26 | 343 | IVb |
| 184 | 10 | *P. deltoides* | II | PdGATA22 | 254 | IVb |
| 185 | 10 | *P. deltoides* | II | PdGATA24 | 254 | IVb |
| 186 | 10 | *P. euphratica* | II | PeGATA21 | 254 | IVb |
| 187 | 10 | *P. euphratica* | II | PeGATA28 | 254 | IVb |
| 188 | 10 | *P. pruinosa* | II | PpGATA19 | 225 | IVb |
| 189 | 10 | *P. tremula* x *alba* | II | PtaaGATA22 | 254 | IVb |
| 190 | 10 | *P. tremula* x *alba* | II | PtaaGATA24 | 254 | IVb |
| 191 | 10 | *P. tremula* | II | PtaGATA22 | 256 | IVb |
| 192 | 10 | *P. trichocarpa* | II | PtrGATA16 | 254 | IVb |
| 193 | 10 | *P. trichocarpa* | II | PtrGATA22 | 254 | IVb |
| 194 | 10 | *P. tremuloides* | II | PtsGATA19 | 254 | IVb |
| 195 | 10 | *P. tremuloides* | II | PtsGATA21 | 254 | IVb |
| 196 | 11 | *P. deltoides* | II | PdGATA20 | 226 | IV4 |
| 197 | 11 | *P. euphratica* | II | PeGATA20 | 238 | IV4 |
| 198 | 11 | *P. pruinosa* | II | PpGATA22 | 235 | IV4 |
| 199 | 11 | *P. tremula* x *alba* | II | PtaaGATA20 | 227 | IV4 |
| 200 | 11 | *P. trichocarpa* | II | PtrGATA10 | 226 | IV4 |
| 201 | 12 | *P. deltoides* | I | PdGATA17 | 212 | IVb |
| 202 | 12 | *P. deltoides* | I | PdGATA6a | 301 | IVb |
| 203 | 12 | *P. deltoides* | I | PdGATA6b | 301 | IVb |
| 204 | 12 | *P. deltoides* | I | PdGATA6c | 295 | IVb |
| 205 | 12 | *P. deltoides* | I | PdGATA6d | 295 | IVb |
| 206 | 12 | *P. deltoides* | I | PdGATA6e | 301 | IVb |
| 207 | 12 | *P. deltoides* | I | PdGATA6f | 232 | IVb |
| 208 | 12 | *P. deltoides* | I | PdGATA6g | 301 | IVb |
| 209 | 12 | *P. deltoides* | I | PdGATA6h | 301 | IVb |
| 210 | 12 | *P. deltoides* | I | PdGATA6i | 295 | IVb |
| 211 | 12 | *P. deltoides* | I | PdGATA7 | 233 | IVb |
| 212 | 12 | *P. euphratica* | I | PeGATA3 | 232 | IVb |
| 213 | 12 | *P. euphratica* | I | PeGATA8a | 314 | IVb |
| 214 | 12 | *P. euphratica* | I | PeGATA8b | 320 | IVb |
| 215 | 12 | *P. pruinosa* | I | PpGATA17 | 187 | IVb |
| 216 | 12 | *P. pruinosa* | I | PpGATA6 | 406 | IVb |
| 217 | 12 | *P. tremula* x *alba* | I | PtaaGATA6 | 302 | IVb |
| 218 | 12 | *P. tremula* x *alba* | I | PtaaGATA7 | 256 | IVb |
| 219 | 12 | *P. tremula* | I | PtaGATA1 | 187 | IVb |
| 220 | 12 | *P. tremula* | I | PtaGATA16 | 316 | IVb |
| 221 | 12 | *P. trichocarpa* | I | PtrGATA12a | 301 | IVb |
| 222 | 12 | *P. trichocarpa* | I | PtrGATA12b | 301 | IVb |
| 223 | 12 | *P. trichocarpa* | I | PtrGATA12c | 301 | IVb |
| 224 | 12 | *P. trichocarpa* | I | PtrGATA12d | 295 | IVb |
| 225 | 12 | *P. trichocarpa* | I | PtrGATA12e | 295 | IVb |
| 226 | 12 | *P. trichocarpa* | I | PtrGATA12f | 235 | IVb |
| 227 | 12 | *P. trichocarpa* | I | PtrGATA12g | 235 | IVb |
| 228 | 12 | *P. trichocarpa* | I | PtrGATA14a | 240 | IVb |
| 229 | 12 | *P. trichocarpa* | I | PtrGATA14b | 236 | IVb |
| 230 | 12 | *P. trichocarpa* | I | PtrGATA14c | 236 | IVb |
| 231 | 12 | *P. trichocarpa* | I | PtrGATA14d | 188 | IVb |
| 232 | 12 | *P. trichocarpa* | I | PtrGATA14e | 188 | IVb |
| 233 | 12 | *P. tremuloides* | I | PtsGATA2 | 187 | IVb |
| 234 | 12 | *P. tremuloides* | I | PtsGATA9 | 384 | IVb |
| 235 | 13 | *P. deltoides* | I | PdGATA11a | 329 | IVb |
| 236 | 13 | *P. deltoides* | I | PdGATA11b | 329 | IVb |
| 237 | 13 | *P. deltoides* | I | PdGATA5 | 327 | IVb |
| 238 | 13 | *P. euphratica* | I | PeGATA11 | 329 | IVb |
| 239 | 13 | *P. euphratica* | I | PeGATA7 | 328 | IVb |
| 240 | 13 | *P. pruinosa* | I | PpGATA10 | 329 | IVb |
| 241 | 13 | *P. pruinosa* | I | PpGATA11 | 328 | IVb |
| 242 | 13 | *P. tremula* x *alba* | I | PtaaGATA12a | 329 | IVb |
| 243 | 13 | *P. tremula* x *alba* | I | PtaaGATA12b | 329 | IVb |
| 244 | 13 | *P. tremula* x *alba* | I | PtaaGATA12c | 329 | IVb |
| 245 | 13 | *P. tremula* x *alba* | I | PtaaGATA5a | 327 | IVb |
| 246 | 13 | *P. tremula* x *alba* | I | PtaaGATA5b | 327 | IVb |
| 247 | 13 | *P. tremula* | I | PtaGATA5a | 327 | IVb |
| 248 | 13 | *P. tremula* | I | PtaGATA5b | 327 | IVb |
| 249 | 13 | *P. tremula* | I | PtaGATA6a | 329 | IVb |
| 250 | 13 | *P. tremula* | I | PtaGATA6b | 329 | IVb |
| 251 | 13 | *P. tremula* | I | PtaGATA6c | 329 | IVb |
| 252 | 13 | *P. trichocarpa* | I | PtrGATA11a | 327 | IVb |
| 253 | 13 | *P. trichocarpa* | I | PtrGATA11b | 327 | IVb |
| 254 | 13 | *P. trichocarpa* | I | PtrGATA27a | 329 | IVb |
| 255 | 13 | *P. trichocarpa* | I | PtrGATA27b | 329 | IVb |
| 256 | 13 | *P. trichocarpa* | I | PtrGATA27c | 329 | IVb |
| 257 | 13 | *P. tremuloides* | I | PtsGATA1 | 329 | IVb |
| 258 | 13 | *P. tremuloides* | I | PtsGATA6 | 327 | IVb |
| 259 | 14 | *P. deltoides* | I | PdGATA9 | 369 | IVb |
| 260 | 14 | *P. euphratica* | I | PeGATA1 | 370 | IVb |
| 261 | 14 | *P. euphratica* | I | PeGATA2 | 331 | IVb |
| 262 | 14 | *P. pruinosa* | I | PpGATA12 | 420 | IVb |
| 263 | 14 | *P. pruinosa* | I | PpGATA5 | 372 | IVb |
| 264 | 14 | *P. tremula* x *alba* | I | PtaaGATA10 | 376 | IVb |
| 265 | 14 | *P. tremula* x *alba* | I | PtaaGATA8 | 333 | IVb |
| 266 | 14 | *P. tremula* | I | PtaGATA14a | 370 | IVb |
| 267 | 14 | *P. tremula* | I | PtaGATA14b | 338 | IVb |
| 268 | 14 | *P. tremula* | I | PtaGATA17a | 330 | IVb |
| 269 | 14 | *P. tremula* | I | PtaGATA17b | 330 | IVb |
| 270 | 14 | *P. trichocarpa* | I | PtrGATA15 | 333 | IVb |
| 271 | 14 | *P. trichocarpa* | I | PtrGATA21 | 376 | IVb |
| 272 | 14 | *P. tremuloides* | I | PtsGATA14 | 333 | IVb |
| 273 | 14 | *P. tremuloides* | I | PtsGATA16 | 370 | IVb |
| 274 | 15 | *P. deltoides* | I | PdGATA13 | 295 | IVb |
| 275 | 15 | *P. deltoides* | I | PdGATA16 | 294 | IVb |
| 276 | 15 | *P. euphratica* | I | PeGATA13 | 294 | IVb |
| 277 | 15 | *P. euphratica* | I | PeGATA6 | 298 | IVb |
| 278 | 15 | *P. pruinosa* | I | PpGATA2 | 298 | IVb |
| 279 | 15 | *P. pruinosa* | I | PpGATA3 | 294 | IVb |
| 280 | 15 | *P. tremula* x *alba* | I | PtaaGATA14 | 295 | IVb |
| 281 | 15 | *P. tremula* x *alba* | I | PtaaGATA17 | 294 | IVb |
| 282 | 15 | *P. tremula* | I | PtaGATA15 | 293 | IVb |
| 283 | 15 | *P. tremula* | I | PtaGATA7 | 294 | IVb |
| 284 | 15 | *P. trichocarpa* | I | PtrGATA31 | 295 | IVb |
| 285 | 15 | *P. trichocarpa* | I | PtrGATA38 | 294 | IVb |
| 286 | 15 | *P. tremuloides* | I | PtsGATA13 | 297 | IVb |
| 287 | 15 | *P. tremuloides* | I | PtsGATA4 | 328 | IVb |
| 288 | 16 | *P. deltoides* | I | PdGATA2 | 306 | IVb |
| 289 | 16 | *P. euphratica* | I | PeGATA10 | 308 | IVb |
| 290 | 16 | *P. pruinosa* | I | PpGATA9 | 308 | IVb |
| 291 | 16 | *P. tremula* x *alba* | I | PtaaGATA2a | 250 | IVb |
| 292 | 16 | *P. tremula* x *alba* | I | PtaaGATA2b | 200 | IVb |
| 293 | 16 | *P. tremula* | I | PtaGATA2a | 307 | IVb |
| 294 | 16 | *P. tremula* | I | PtaGATA2b | 176 | IVb |
| 295 | 16 | *P. trichocarpa* | I | PtrGATA3 | 200 | IVb |
| 296 | 16 | *P. tremuloides* | I | PtsGATA8 | 194 | IVb |
| 297 | 17 | *P. deltoides* | I | PdGATA15 | 380 | IVb |
| 298 | 17 | *P. deltoides* | I | PdGATA8 | 373 | IVb |
| 299 | 17 | *P. euphratica* | I | PeGATA15 | 380 | IVb |
| 300 | 17 | *P. euphratica* | I | PeGATA17 | 373 | IVb |
| 301 | 17 | *P. pruinosa* | I | PpGATA14 | 380 | IVb |
| 302 | 17 | *P. pruinosa* | I | PpGATA7 | 373 | IVb |
| 303 | 17 | *P. tremula* x *alba* | I | PtaaGATA16a | 380 | IVb |
| 304 | 17 | *P. tremula* x *alba* | I | PtaaGATA16b | 264 | IVb |
| 305 | 17 | *P. tremula* x *alba* | I | PtaaGATA16c | 353 | IVb |
| 306 | 17 | *P. tremula* x *alba* | I | PtaaGATA18 | 354 | IVb |
| 307 | 17 | *P. tremula* x *alba* | I | PtaaGATA9a | 373 | IVb |
| 308 | 17 | *P. tremula* x *alba* | I | PtaaGATA9b | 357 | IVb |
| 309 | 17 | *P. tremula* | I | PtaGATA10a | 386 | IVb |
| 310 | 17 | *P. tremula* | I | PtaGATA10b | 380 | IVb |
| 311 | 17 | *P. tremula* | I | PtaGATA4 | 373 | IVb |
| 312 | 17 | *P. trichocarpa* | I | PtrGATA20 | 373 | IVb |
| 313 | 17 | *P. trichocarpa* | I | PtrGATA36 | 380 | IVb |
| 314 | 17 | *P. trichocarpa* | I | PtrGATA39 | 210 | IVb |
| 315 | 17 | *P. tremuloides* | I | PtsGATA15 | 375 | IVb |
| 316 | 17 | *P. tremuloides* | I | PtsGATA7 | 380 | IVb |
| 317 | 18 | *P. deltoides* | I | PdGATA14 | 231 | IVb |
| 318 | 18 | *P. euphratica* | I | PeGATA5 | 247 | IVb |
| 319 | 18 | *P. pruinosa* | I | PpGATA1 | 250 | IVb |
| 320 | 18 | *P. tremula* x *alba* | I | PtaaGATA15 | 250 | IVb |
| 321 | 18 | *P. tremula* | I | PtaGATA3a | 246 | IVb |
| 322 | 18 | *P. tremula* | I | PtaGATA3b | 246 | IVb |
| 323 | 18 | *P. tremula* | I | PtaGATA3c | 246 | IVb |
| 324 | 18 | *P. tremula* | I | PtaGATA3d | 246 | IVb |
| 325 | 18 | *P. trichocarpa* | I | PtrGATA32 | 251 | IVb |
| 326 | 18 | *P. tremuloides* | I | PtsGATA11 | 246 | IVb |
| 327 | 19 | *P. deltoides* | I | PdGATA3 | 246 | IVb |
| 328 | 19 | *P. euphratica* | I | PeGATA16 | 246 | IVb |
| 329 | 19 | *P. pruinosa* | I | PpGATA13 | 266 | IVb |
| 330 | 19 | *P. tremula* x *alba* | I | PtaaGATA3 | 246 | IVb |
| 331 | 19 | *P. tremula* | I | PtaGATA12 | 246 | IVb |
| 332 | 19 | *P. trichocarpa* | I | PtrGATA6 | 246 | IVb |
| 333 | 19 | *P. tremuloides* | I | PtsGATA12 | 244 | IVb |
| 334 | 20 | *P. deltoides* | I | PdGATA1a | 270 | IVb |
| 335 | 20 | *P. deltoides* | I | PdGATA1b | 258 | IVb |
| 336 | 20 | *P. deltoides* | I | PdGATA1c | 258 | IVb |
| 337 | 20 | *P. deltoides* | I | PdGATA1d | 258 | IVb |
| 338 | 20 | *P. deltoides* | I | PdGATA1e | 258 | IVb |
| 339 | 20 | *P. deltoides* | I | PdGATA4 | 258 | IVb |
| 340 | 20 | *P. euphratica* | I | PeGATA12 | 256 | IVb |
| 341 | 20 | *P. euphratica* | I | PeGATA14 | 256 | IVb |
| 342 | 20 | *P. euphratica* | I | PeGATA18 | 258 | IVb |
| 343 | 20 | *P. pruinosa* | I | PpGATA15 | 258 | IVb |
| 344 | 20 | *P. pruinosa* | I | PpGATA8 | 258 | IVb |
| 345 | 20 | *P. tremula* x *alba* | I | PtaaGATA1 | 336 | IVb |
| 346 | 20 | *P. tremula* x *alba* | I | PtaaGATA4a | 259 | IVb |
| 347 | 20 | *P. tremula* x *alba* | I | PtaaGATA4b | 258 | IVb |
| 348 | 20 | *P. tremula* | I | PtaGATA11a | 258 | IVb |
| 349 | 20 | *P. tremula* | I | PtaGATA11b | 258 | IVb |
| 350 | 20 | *P. tremula* | I | PtaGATA9a | 259 | IVb |
| 351 | 20 | *P. tremula* | I | PtaGATA9b | 258 | IVb |
| 352 | 20 | *P. trichocarpa* | I | PtrGATA1 | 336 | IVb |
| 353 | 20 | *P. trichocarpa* | I | PtrGATA9 | 258 | IVb |
| 354 | 20 | *P. tremuloides* | I | PtsGATA10 | 258 | IVb |
| 355 | 20 | *P. tremuloides* | I | PtsGATA3a | 259 | IVb |
| 356 | 20 | *P. tremuloides* | I | PtsGATA3b | 258 | IVb |
| 357 | 21 | *P. deltoides* | I | PdGATA10 | 354 | IVb |
| 358 | 21 | *P. deltoides* | I | PdGATA12 | 352 | IVb |
| 359 | 21 | *P. euphratica* | I | PeGATA4 | 358 | IVb |
| 360 | 21 | *P. euphratica* | I | PeGATA9a | 355 | IVb |
| 361 | 21 | *P. euphratica* | I | PeGATA9b | 355 | IVb |
| 362 | 21 | *P. pruinosa* | I | PpGATA16 | 358 | IVb |
| 363 | 21 | *P. pruinosa* | I | PpGATA4 | 355 | IVb |
| 364 | 21 | *P. tremula* x *alba* | I | PtaaGATA11a | 354 | IVb |
| 365 | 21 | *P. tremula* x *alba* | I | PtaaGATA11b | 354 | IVb |
| 366 | 21 | *P. tremula* x *alba* | I | PtaaGATA11c | 354 | IVb |
| 367 | 21 | *P. tremula* x *alba* | I | PtaaGATA13a | 352 | IVb |
| 368 | 21 | *P. tremula* x *alba* | I | PtaaGATA13b | 352 | IVb |
| 369 | 21 | *P. tremula* | I | PtaGATA13a | 352 | IVb |
| 370 | 21 | *P. tremula* | I | PtaGATA13b | 352 | IVb |
| 371 | 21 | *P. tremula* | I | PtaGATA13c | 352 | IVb |
| 372 | 21 | *P. tremula* | I | PtaGATA8a | 354 | IVb |
| 373 | 21 | *P. tremula* | I | PtaGATA8b | 354 | IVb |
| 374 | 21 | *P. tremula* | I | PtaGATA8c | 354 | IVb |
| 375 | 21 | *P. tremula* | I | PtaGATA8d | 354 | IVb |
| 376 | 21 | *P. trichocarpa* | I | PtrGATA25a | 354 | IVb |
| 377 | 21 | *P. trichocarpa* | I | PtrGATA25b | 354 | IVb |
| 378 | 21 | *P. trichocarpa* | I | PtrGATA25c | 354 | IVb |
| 379 | 21 | *P. trichocarpa* | I | PtrGATA29a | 446 | IVb |
| 380 | 21 | *P. trichocarpa* | I | PtrGATA29b | 352 | IVb |
| 381 | 21 | *P. trichocarpa* | I | PtrGATA29c | 352 | IVb |
| 382 | 21 | *P. tremuloides* | I | PtsGATA17a | 354 | IVb |
| 383 | 21 | *P. tremuloides* | I | PtsGATA17b | 354 | IVb |
| 384 | 21 | *P. tremuloides* | I | PtsGATA5 | 352 | IVb |
| 385 | Orphan | *P. deltoides* | I | PdGATA18 | 82 | IVp |
| 386 | Orphan | *P. euphratica* | II | PeGATA19 | 383 | IV4 |
| 387 | Orphan | *P. euphratica* | II | PeGATA23 | 147 | IV4 |
| 388 | Orphan | *P. pruinosa* | II | PpGATA21 | 609 | IV4 |
| 389 | Orphan | *P. pruinosa* | II | PpGATA23 | 486 | IVp |
