# Supplementary material for: Genome-wide comparative analyses of GATA transcription factors among seven Populus genomes
Source: Sci Rep. 2021 Aug 16;11:16578. doi: 10.1038/s41598-021-95940-5 (PMC8367991; doi:10.1038/s41598-021-95940-5)
Supplement: Supplementary file 13 — Supplementary Information 13. [file 41598_2021_95940_MOESM13_ESM.docx]

**Table S8.** Characteristics of 21 *Populus* GATA gene clusters (PCs)

| **PCs** | **# of species** | ***Populus trichocarpa*** | ***Populus pruinosa*** | ***Populus euphratica*** | ***Populus deltoides*** | ***Populus tremuloides*** | ***Populus tremula*** | ***Populus tremula* x *alba*** |
| --- | --- | --- | --- | --- | --- | --- | --- | --- |
| PC01 | 7 | 4 | 4 | 4 | 4 | 4 | 4 | 4 |
| PC02 | 7 | 2 | 1 | 1 | 2 | 2 | 1 | 1 |
| PC03 | 7 | 2 | 1 | 2 | 2 | 2 | 2 | 2 |
| PC04 | 7 | 1 | 1 | 1 | 1 | 1 | 1 | 1 |
| PC05 | 7 | 2 | 2 | 2 | 2 | 2 | 1 | 2 |
| PC06 | 7 | 2 | 2 | 2 | 2 | 2 | 2 | 2 |
| PC07 | 7 | 1 | 1 | 1 | 1 | 1 | 1 | 1 |
| PC08 | 7 | 2 | 2 | 2 | 2 | 2 | 2 | 2 |
| PC09 | 7 | 2 | 2 | 2 | 1 | 2 | 1 | 2 |
| PC10 | 7 | 2 | 1 | 2 | 2 | 2 | 1 | 2 |
| PC11 | 5 | 1 | 1 | 1 | 1 | 0 | 0 | 1 |
| PC12 | 7 | 2 | 2 | 2 | 3 | 2 | 2 | 2 |
| PC13 | 7 | 2 | 2 | 2 | 2 | 2 | 2 | 2 |
| PC14 | 7 | 2 | 2 | 2 | 1 | 2 | 2 | 2 |
| PC15 | 7 | 2 | 2 | 2 | 2 | 2 | 2 | 2 |
| PC16 | 7 | 1 | 1 | 1 | 1 | 1 | 1 | 1 |
| PC17 | 7 | 3 | 2 | 2 | 2 | 2 | 2 | 3 |
| PC18 | 7 | 1 | 1 | 1 | 1 | 1 | 1 | 1 |
| PC19 | 7 | 1 | 1 | 1 | 1 | 1 | 1 | 1 |
| PC20 | 7 | 2 | 2 | 3 | 2 | 2 | 2 | 2 |
| PC21 | 7 | 2 | 2 | 2 | 2 | 2 | 2 | 2 |
| **Total** |  | **39** | **35** | **38** | **37** | **37** | **33** | **38** |
