# Supplementary material for: Genome-wide comparative analyses of GATA transcription factors among seven Populus genomes
Source: Sci Rep. 2021 Aug 16;11:16578. doi: 10.1038/s41598-021-95940-5 (PMC8367991; doi:10.1038/s41598-021-95940-5)
Supplement: Supplementary file 14 — Supplementary Information 14. [file 41598_2021_95940_MOESM14_ESM.docx]

**Table S9.** List of RNA-Seq raw reads used for analyzing expression level of GATA TFs

| ***Populus* species name** | **Tissue** | **NCBI Accessions** | **# of reads** | **Total bases (bp)** |
| --- | --- | --- | --- | --- |
| *Populus deltoides* | Leaf | SRR8554758 | 50,372,646 | 7,606,269,546 |
|  | Phloem | SRR8840177 | 30,846,146 | 4,657,768,046 |
|  | Xylem | SRR10433244 | 50,564,918 | 4,566,963,368 |
|  | Root | SRR8840190 | 34,987,008 | 5,283,038,208 |
| *Populus pruinosa* | Leaf | SRR6024998 | 246,980,290 | 30,872,536,250 |
|  |  | SRR6025028 | 278,878,500 | 34,859,812,500 |
|  |  | SRR6025029 | 282,250,940 | 35,281,367,500 |
|  | Phloem | SRR6025179 | 247,510,462 | 30,938,807,750 |
|  |  | SRR6025129 | 276,733,894 | 34,591,736,750 |
|  |  | SRR6025130 | 263,982,536 | 32,997,817,000 |
|  | Xylem | SRR6025193 | 274,607,258 | 34,325,907,250 |
|  |  | SRR6025194 | 279,194,954 | 34,899,369,250 |
|  |  | SRR6025195 | 274,721,664 | 34,340,208,000 |
|  | Root | SRR6025196 | 291,778,630 | 36,472,328,750 |
|  |  | SRR6025197 | 287,974,064 | 35,996,758,000 |
|  |  | SRR6025198 | 267,576,274 | 33,447,034,250 |
