# Supplementary material for: Genome-wide comparative analyses of GATA transcription factors among seven Populus genomes
Source: Sci Rep. 2021 Aug 16;11:16578. doi: 10.1038/s41598-021-95940-5 (PMC8367991; doi:10.1038/s41598-021-95940-5)
Supplement: Supplementary file 15 — Supplementary Information 15. [file 41598_2021_95940_MOESM15_ESM.docx]

**Table S10.** List of GATA TFs having TMHs predicted by TMHMM 2.0

| ***Populus* species name** | **GATA TFs name** | **# of TMHs** |
| --- | --- | --- |
| *Populus trichocarpa* | PtrGATA14b | 1 |
|  | PtrGATA14c | 1 |
| *Populus pruinosa* | PpGATA21 | 1 |
|  | PpGATA25 | 1 |
| *Populus tremula* x *alba* | PtaaGATA23 | 1 |
| **Total** |  | **5** |
